# Supplementary material for: A tropical geometry for bounded biochemical state spaces
Source: Patterns (N Y). 2026 Jul 9;7(10):101613. doi: 10.1016/j.patter.2026.101613 (PMC13674305; doi:10.1016/j.patter.2026.101613)
Supplement: Supplementary file 1 [file mmc1.pdf]

Patterns, Volume *7*

Supplemental information

A tropical geometry for bounded biochemical state spaces

James N. Cobley

Supplemental Notes

Supplemental Note 1 — Formal structure of bounded biochemical state spaces

S1.1. State space and observables

Let a biochemical modification state be represented by a bounded occupancy

$$x \in \mathcal{X} := [0,1],$$

where $x = 0$denotes fully unmodified (e.g., fully reduced) and $x = 1$ denotes fully modified (e.g., fully oxidised). For proteoform-level objects comprising $r$sites, we consider the product space

$$\mathcal{X}^{(r)} := [0,1]^r.$$

In many PTM contexts, the measured quantity is an ensemble occupancy (fraction modified), and therefore a coarse-grained observable. We model this via a measurement map

$$M: \Omega \to \mathcal{X},$$

from a (typically unobserved) space of micro-trajectories Ω to a bounded observable x.

S1.2. Hard bounds and closure

The state space $\mathcal{X}$ is closed and bounded. Any operation interpreted as a "translation" $x \mapsto x + \delta$with $\delta \neq 0$ is not closed on $\mathcal{X}$ in general, since $x + \delta \notin [0,1]$ whenever x is sufficiently close to a boundary.

A natural way to represent finite changes while preserving closure is by *saturated* (clipped) maps. For example, define oxidation and reduction updates of magnitude $a, b \geq 0$ by

$$\mathrm{Ox}_a(x) := \min(1, x + a), \mathrm{Red}_b(x) := \max(0, x - b).$$

Then $\mathrm{Ox}_a, \mathrm{Red}_b: \mathcal{X} \to \mathcal{X}$ are well-defined endomorphisms.

S1.3. Absorbing boundaries

Definition (absorbing boundary). A boundary point $x^\star \in \{0,1\}$ is absorbing for an update family $\{F_\theta\}$ if for all admissible θ in a direction,

$$F_\theta(x^\star) = x^\star.$$

Proposition S1 (absorption under saturation). For all $a \geq 0$and $b \geq 0$,

$$\mathrm{Ox}_a(1) = 1, \mathrm{Red}_b(0) = 0.$$

Hence 1 is absorbing for oxidation updates and 0 is absorbing for reduction updates.

Proof. Immediate from the definitions: $\min(1, 1 + a) = 1$ and $\max(0, 0 - b) = 0$. □

S1.4. Many-to-one mapping and non-invertibility

Proposition S2 (many-to-one collapse). For any $a > 0$,

$$\mathrm{Ox}_a(x) = 1 \text{ for all } x \in [1 - a, 1].$$

Similarly, for any $b > 0$,

$$\mathrm{Red}_b(x) = 0 \text{ for all } x \in [0, b].$$

Proof. If $x \geq 1 - a$, then $x + a \geq 1$ so $\min(1, x + a) = 1$. The reduction case is analogous. □

Corollary S2.1 (non-invertibility). For any $a > 0$, Ox_a is not injective (hence not invertible) on $\mathcal{X}$. Likewise, Red_b for any $b > 0$.

This formalises the claim that distinct biochemical histories can collapse onto the same observed boundary state.

S1.5. Equal-magnitude changes encode unequal meaning (position dependence)

For a fixed increment $\delta > 0$, the *available capacity* to move upward without saturation at state x is $c_+(x) := 1 - x$; downward is $c_-(x) := x$. A change of magnitude δ is therefore interpreted relative to state-dependent capacity.

Proposition S3 (position dependence). For $\delta > 0$, the "same" increment δ produces a saturated outcome iff $x \geq 1 - \delta$. Hence, the effect of a δ-update depends on x through $c_+(x)$.

This captures formally why a shift $0 \to 0.1$ and $0.5 \to 0.6$ are not equivalent: only the former crosses the absorbing neighbourhood where collapse is common and capacity is minimal.

S1.6. Proteoform ensembles induce intrinsic many-to-one structure

Let r binary sites define a proteoform state space $\{0,1\}^r$. A proteoform ensemble is a distribution p on $\{0,1\}^r$, i.e. $p \in \Delta_{2^r}$ (the probability simplex). The measured site occupancies are the marginals

$$m_i = \mathbb{E}_p[s_i], i = 1, \dots, r,$$

defining a mapping

$$\pi: \Delta_{2^r} \to [0,1]^r, \pi(p) = (m_1, \dots, m_r).$$

Proposition S4 (non-invertibility of marginalisation). For $r \geq 2$, the map π is generically many-to-one: distinct proteoform distributions can yield identical marginal occupancies.

Proof (constructive). For $r = 2$, let $p^{(1)}$ place mass $(1/2, 0, 0, 1/2)$ on $(00, 01, 10, 11)$ and let $p^{(2)}$ place mass $(0, 1/2, 1/2, 0)$. Both yield marginals $m_1 = m_2 = 1/2$ but $p^{(1)} \neq p^{(2)}$. The general case follows by embedding this 2-site construction within r sites. □

This shows that many-to-one structure is present even before considering dynamics: it is intrinsic to coarse-grained measurement of proteoform ensembles.

Supplemental Note 2 — Incompatibility of bounded, absorbing state spaces with linear/Euclidean representations

This note formalises the sense in which linear and Euclidean frameworks assume properties (global translation, invertibility, symmetry) that are violated by bounded, absorbing state spaces.

S2.1. Linear structure implies global translation and inverses

A real vector space $(V, +)$ is an abelian group under addition: for every $v \in V$, an additive inverse $-v$ exists with $v + (-v) = 0$, and translation maps $T_u(v) = v + u$ are bijections of V.

By contrast, bounded biochemical state spaces $\mathcal{X} = [0,1]$ do not admit a globally closed translation operation $x \mapsto x + \delta$ for arbitrary $\delta \neq 0$. Any attempt to enforce closure by saturation introduces many-to-one collapse near boundaries (Proposition S2), which destroys bijectivity and therefore invertibility.

S2.2. No globally invertible "translation-like" dynamics on [0,1] with absorption

Proposition S5 (absorption implies non-bijectivity). Let $F: \mathcal{X} \to \mathcal{X}$ be any map such that there exists an interval $I \subseteq \mathcal{X}$ with $F(x) = x^\star$ for all $x \in I$ (collapse to an absorbing state). Then F is not injective and has no inverse on $\mathcal{X}$.

Proof. If $| I | > 1$, pick $x \neq y \in I$; then $F(x) = F(y) = x^\star$, so F is not injective. □

Because absorption/collapse is an intrinsic feature of the object (Supplemental Note 1), any representation that assumes invertible dynamics is categorically mismatched.

S2.3. Euclidean distance is translation-invariant; bounded saturation is not

In Euclidean spaces, distances satisfy translation invariance:

$$\| (v + u) - (w + u) \| = \| v - w \| \; \forall u, v, w.$$

In bounded state spaces with saturation, the effect of applying the "same" increment depends on position (Proposition S3), so translation-invariance fails at the level of dynamics. Consequently, Euclidean interpretations of "equal distances imply equal meaning" are not valid for state evolution on [0,1] in the presence of absorbing boundaries.

Supplemental Note 3 | Tropical algebra as a natural realisation of the requirements of bounded biochemical state spaces

Supplemental Notes 1 and 2 established that bounded biochemical state spaces possess hard boundaries, absorbing states, many-to-one ensemble mappings, and generically non-invertible dynamics, and that these properties are categorically incompatible with linear and Euclidean representations. This note addresses the natural follow-up question: why tropical algebra, rather than other boundary-aware mathematical approaches?

We do not claim that tropical algebra is the unique resolution to the categorical mismatch problem. Rather, we argue that it is a natural realisation of the minimal algebraic requirements imposed by the object, and that this naturalness can be understood on both algebraic and physical grounds.

S3.1. Minimal requirements revisited

From Supplemental Notes 1 and 2, any algebraic framework suitable for bounded biochemical state spaces must:

(i) respect hard boundaries without relying on unconstrained translation;

(ii) accommodate absorbing states and many-to-one mappings without assuming global invertibility;

(iii) represent irreversible, path-dependent transitions without assuming symmetry;

(iv) remain well-defined on discrete state spaces such as proteoform ensembles.

These requirements are imposed by the object itself and are not negotiable for a categorically correct representation.

S3.2. How alternative boundary-aware approaches fare

Several established approaches handle bounded data and are worth considering as alternatives.

Supplementary Table 1.

Approach	(i) Hard boundaries intrinsic	(ii) Non-invertibility preserved	(iii) Discrete state spaces	(iv) Invertibility restored by construction
**Linear / Euclidean** e.g. PCA, linear regression	No	No	No	Yes
**Logit transformation**	Partial*	No	No	Yes

Approach	(i) Hard boundaries intrinsic	(ii) Non-invertibility preserved	(iii) Discrete state spaces	(iv) Invertibility restored by construction
**Beta regression**	Partial*	No	No	Yes
**Compositional data analysis** Aitchison / log-ratio	Partial*	No	No	Yes
**Bounded tropical semiring** this work	Yes	Yes	Yes	No

*These approaches acknowledge that data are bounded but handle this by mapping observations into an unbounded space (e.g. the real line or the simplex with log-ratio coordinates) prior to applying standard linear methods. Boundaries are managed by transformation rather than respected intrinsically. Invertibility is restored by construction, concealing the non-invertibility that is a structural property of the biological object (Proposition S2, S5).

S3.3. Algebraic grounds for the naturalness of tropical algebra

Tropical algebra replaces standard addition with the max operation and standard multiplication with capped addition (min(1, a + b) in the bounded variant used here). These operations satisfy requirements (i)-(iv) directly:

- Boundaries are intrinsic: the tropical operations are defined to be closed on [0,1] without external clamping.

- Absorbing states arise structurally: once a state reaches a boundary, further operations in the saturating direction leave it unchanged, reflecting biological saturation as an algebraic property rather than a post-hoc correction.

- Non-invertibility is preserved: many-to-one collapse at boundaries is a consequence of the operations themselves (consistent with Propositions S2 and S5), not a failure to be corrected.

- Discrete state spaces are handled directly: tropical operations are well-defined on finite sets and do not require smooth manifolds or infinitesimal neighbourhoods.

S3.4. Physical grounds for the naturalness of tropical algebra

Beyond algebraic convenience, two features of tropical algebra correspond to physical principles in a way that alternative approaches do not.

First, the tropical min operation — central to min-plus tropical algebra and implicit in the path-dependence structure of the bounded semiring — is physically consistent with the principle of least action. In physical systems, nature selects the path of minimum cost among admissible trajectories. The tropical min selects the minimum among competing values,

encoding path optimality as an algebraic primitive. This correspondence means the framework resonates with how physical systems actually evolve rather than imposing a representational convenience.

Second, tropical algebra is idempotent under the max operation: a ⊕ a = a. This means applying the same operation twice produces no additional change — which is physically exact for a saturated or boundary-proximal biochemical site. A fully oxidised cysteine subjected to further oxidation remains fully oxidised; a fully phosphorylated residue cannot be further phosphorylated. In linear algebra, this behaviour requires external enforcement (clamping); in tropical algebra it is a structural consequence of the operations. Idempotency therefore encodes the physical reality of saturation without additional assumptions.

Together, these algebraic and physical correspondences support the choice of tropical algebra as a natural realisation of the requirements of bounded biochemical state spaces, without requiring the claim that it is the only such realisation.

Supplemental Note 4 | A bounded tropical semiring for biochemical occupancies

S3.1. Definition (bounded tropical operations)

Let $\mathbb{T}_{[0,1]} := ([0,1], \oplus, \otimes)$ with operations

$$a \oplus b := \max(a, b),$$
$$a \otimes b := \min(1, a + b).$$

(Here ⊗ is *capped addition*, ensuring closure on [0,1].)

S3.2. Algebraic properties

Proposition S6 (closure). For all $a, b \in [0,1]$, $a \oplus b \in [0,1]$and $a \otimes b \in [0,1]$.

Proof. $\max(a, b) \in [0,1]$. Also $a + b \in [0,2]$so $\min(1, a + b) \in [0,1]$. □

Proposition S7 (commutativity and associativity). ⊕ and ⊗ are commutative and associative on [0,1].

Proof. Commutativity is immediate. Associativity of ⊕ follows from associativity of max . For ⊗,

$$(a \otimes b) \otimes c = \min(1, \min(1, a + b) + c) = \min(1, a + b + c) = a \otimes (b \otimes c).$$

□

Proposition S8 (identities and absorption). The element 0is the identity for both operations:

$$a \oplus 0 = a, a \otimes 0 = a,$$

and 1is absorbing for $\otimes$:

$$a \otimes 1 = 1.$$

Proof. Immediate from definitions. □

This mirrors biochemical structure: 1acts as saturation (absorbing under further oxidation-like composition).

Proposition S9 (idempotency and non-existence of additive inverses). $\oplus$is idempotent: $a \oplus a = a$. Consequently, $([0,1], \oplus)$ cannot be a group and admits no global inverses compatible with $\oplus$.

Proof. $a \oplus a = \max(a, a) = a$. If $\oplus$ had inverses, idempotency would force $a = 0$for all a, a contradiction. □

This provides a clean algebraic statement of non-invertibility without appealing to dynamics.

Supplemental Note 5 | Order dependence and non-commutativity of biochemical transition sequences

The main text distinguishes commutativity of tropical operations from order dependence of *state transition sequences*. We formalise that distinction here using oxidation/reduction operators on [0,1].

S4.1. State transition operators

Define oxidation and reduction operators as in S1:

$$\mathrm{Ox}_a(x) = \min(1, x + a), \mathrm{Red}_b(x) = \max(0, x - b).$$

These maps are monotone and closed on $[0, 1]$.

S4.2. Non-commutativity of mixed transition sequences

Proposition S10 (order dependence). There exist $a, b > 0$ and $x \in [0,1]$ such that

$$(\mathrm{Ox}_a \circ \mathrm{Red}_b)(x) \neq (\mathrm{Red}_b \circ \mathrm{Ox}_a)(x).$$

Proof (constructive). Choose $x = 1 - \varepsilon$ with $0 < \varepsilon < a$, and choose $b > a - \varepsilon$. Then

$$(\mathrm{Ox}_a \circ \mathrm{Red}_b)(x) = \mathrm{Ox}_a(1 - \varepsilon - b) = \min(1, 1 - \varepsilon - b + a),$$

while

$$(\mathrm{Red}_b \circ \mathrm{Ox}_a)(x) = \mathrm{Red}_b(1) = \max(0,1 - b) = 1 - b.$$

With the above choices, these quantities differ (one path saturates before reduction, the other reduces after saturation). □

This shows that although $\oplus$ is commutative, *mixed biochemical update sequences* need not commute because boundaries change the effect of subsequent operations.

S4.3. Path dependence via collapse

Proposition S11 (history erasure near boundaries). For any $a > 0$, the set $[1 - a, 1]$is collapsed by $\mathrm{Ox}_a$to the single value 1. Therefore, trajectories entering this region become indistinguishable under subsequent observations.

This formalises "irretrievable loss of history" at the observation level and explains why ensemble dynamics are physically non-invertible.

Supplemental Note 6 | Mathematical construction of Oxi-Shapes

Bounded biochemical state space

Let $\mathcal{L} = \{1, \dots, N\}$ denote a finite index set corresponding to invariant molecular identities (here, cysteine residues). For each site $i \in \mathcal{L}$, an experimental measurement assigns a scalar oxidation occupancy

$$x_i \in [0,1],$$

representing the fraction of molecules observed in an oxidised state at that site. The unit interval constitutes a **bounded biochemical state space** with absorbing boundaries at 0 (fully reduced) and 1 (fully oxidised).

Measurement-native object construction

An Oxi-Shape is defined as a mapping

$$f: \mathcal{L} \to [0,1], i \mapsto x_i,$$

assigning a bounded scalar state to each invariant site identity.

To visualise this object, sites are embedded in a discrete lattice index space by assigning each $i \in \mathcal{L}$a unique and invariant pair of coordinates (u_i, v_i), forming a two-dimensional discrete lattice. This embedding is **index-based only** and does not encode biological proximity, similarity, or interaction.

The measured oxidation value x_i is represented as a bounded scalar deformation along a third axis. The resulting object is a **high-dimensional discrete lattice with a bounded scalar field**, fully determined by experimental measurements.

At this stage, the object carries **no topology, adjacency, metric, or relational structure** beyond site identity and bounded state assignment.

Algebraic constraints of bounded state spaces

Because $x_i \in [0,1]$, the state space is not closed under unconstrained linear operations. In particular:

- Translation invariance does not hold.
- Linear combinations can generate invalid states outside [0,1].
- Equal numerical differences do not correspond to equal physical changes near boundaries.
- Global invertibility and symmetry assumptions underlying Euclidean vector spaces are violated.

As a consequence, applying linear projections, Euclidean distances, or variance-maximising operators to bounded biochemical occupancies is **categorically incompatible** with the data-generating process.

Oxi-Shapes therefore adopts a **bounded (tropical) algebraic perspective**, in which admissible operations must respect the absorbing boundaries and non-linearity intrinsic to the state space.

4 Information conservation and state occupancy

In Oxi-Shapes, each site i carries a state variable $x_i$that is **mutable across conditions** yet **conserved at the level of information**: site identity is invariant, and whenever a site is observed it is assigned a well-defined bounded value.

There are no null internal states within the measurement space; absence of information arises only from missing observations, not from the algebra of the state space itself.

Corollary.
This construction enables direct information-theoretic interpretation of biochemical state without logarithmic transforms, binning procedures, or assumptions of unboundedness. Measures of order, disorder, and entropy may therefore be defined directly on the bounded object once a representation consistent with the algebra is established.

S1.5 Scope and non-assumptions

The construction above is intentionally minimal. At this stage, Oxi-Shapes does **not** assume:

- temporal dynamics or trajectories,
- kinetic or mechanistic models,
- neighbourhood relations between sites,

- smoothness or continuity,
- biological interaction networks.

All subsequent geometric, energetic, or relational structures are introduced only after this measurement-native object has been defined.

Supplementary Note 7 | Volume and configurational degeneracy in bounded cysteine state space

Definition of the bounded cysteine state space

Consider a protein system with *N* cysteine sites, each of which can occupy a reduced or oxidised state. In Oxi-Shapes, each site *i* is associated with a bounded scalar oxidation value

$$x_i \in [0,1],$$

representing the fraction of molecules in which that cysteine is oxidised at the time of measurement.

The admissible biochemical state space is therefore bounded and discrete, with each site contributing exactly one scalar degree of freedom. The full configuration of the system at a given observational snapshot is represented by the vector

$$\mathbf{x} = (x_1, x_2, \dots, x_N).$$

Volume as a geometric invariant

The total volume *V* of an Oxi-Shapes object is defined as the arithmetic mean of site-wise oxidation values:

$$V = \frac{1}{N}\sum_{i=1}^{N} x_i \,.$$

Geometrically, this quantity corresponds to the average uplift of the discrete lattice along the bounded oxidation axis. Because each site contributes exactly one bounded scalar, the volume is invariant under lattice permutation and independent of site ordering.

Importantly, no transformation, normalisation, or rescaling is applied beyond the intrinsic unit interval already encoded in the data. By construction, this volume is numerically identical to the arithmetic mean oxidation, but it is here interpreted as a geometric invariant of the lattice.

Internal redox entropy as an order–disorder coordinate

We define **internal redox entropy** as this volume coordinate *V*. This quantity provides a measure of order–disorder on the bounded cysteine lattice:

- **Maximum internal order** occurs when all sites occupy the same boundary state (all 0 or all 1), corresponding to a perfectly flat lattice with minimal configurational freedom.
- **Maximum internal disorder** occurs at intermediate occupancy, where reduced and oxidised states are most evenly distributed.

Crucially, this definition makes no claim about thermodynamic entropy, heat exchange, dissipation, or microscopic irreversibility. Internal redox entropy is a *geometric projection* of configurational structure defined on a bounded biochemical state space at a fixed observational snapshot.

Configurational degeneracy of the mean

While the volume *V* uniquely specifies the mean oxidation, it does **not** uniquely specify the underlying configuration. For a system of *N* binary sites, the number of distinct configurations consistent with a given mean oxidation is given by the binomial coefficient:

$$\Omega(N, k) = \binom{N}{k},$$

where

$$k = N \times V.$$

This quantity measures the **configurational degeneracy** associated with a given volume coordinate. As *V* varies from 0 to 1, the degeneracy exhibits a characteristic inverted-U structure, with:

- minimal degeneracy at the boundaries (*V* = 0 or 1),
- maximal degeneracy at *V* = 0.5.

This structure reflects a fundamental property of bounded binary state spaces and is independent of any biological assumptions.

Interpretation of realised configurations

The observed Oxi-Shapes lattice corresponds to one realised configuration among the Ω admissible configurations consistent with the measured mean oxidation. The lattice visualisation therefore specifies **which configuration occurred**, while the degeneracy curve quantifies **how many alternative configurations were mathematically possible but not realised**.

In this sense, internal redox entropy decomposes naturally into:

- a **real component** (the volume / mean oxidation), and
- a **configurational context** (the degeneracy associated with that mean).

This separation clarifies how small changes in mean oxidation can correspond to disproportionately large changes in the size of the admissible configuration space, particularly near the boundaries of the bounded state space.

Scope and limitations

This construction is explicitly mesoscopic and non-invertible. Multiple biochemical histories may map to the same observed configuration, and no claims are made regarding the dynamics, kinetics, or external entropy production that gave rise to the measured state.

Rather, Supplemental Note 2 establishes a mathematically complete and bounded framework for interpreting cysteine redox measurements as geometric objects with well-defined volume and configurational structure.

Supplemental Note 8 | Laplacian curvature and derived energy functionals on the bounded lattice

Let $\phi \in [0,1]^N$ denote the bounded occupancy field defined on a discrete index lattice of N sites. A minimal 4-neighbour adjacency is imposed on the lattice, yielding a graph $G = (V, E)$ with $|V| = N$. Let A denote the adjacency matrix and D the diagonal degree matrix. The (unnormalised) graph Laplacian is defined as

$$L = D - A.$$

We define Laplacian curvature on the lattice as the action of the graph Laplacian on the bounded field,

$$\kappa \equiv L\phi,$$

which constitutes a discrete second-difference operator measuring local variation of the scalar field relative to its neighbours.

A quadratic variation energy functional (Dirichlet energy) is defined by

$$E_D(\phi) \equiv \frac{1}{2} \sum_{(i,j)\in E} (\phi_i - \phi_j)^2 \; = \; \frac{1}{2} \phi^\top L \phi.$$

This quantity measures the total squared variation of the field across adjacent lattice indices and may be expressed as a per-node energy density by distributing edge contributions equally to incident nodes.

To encode an explicit ground-state bias at $\phi = 0$, we define a bounded Morse-type potential at each lattice site, parameterised by $a > 0$,

$$V(\phi_i) = (1 - e^{-a\phi_i})^2.$$

The resulting **Morse energy functional** is summarised either as the mean

$$\frac{1}{N}\sum_{i=1}^{N} V(\phi_i)$$

or as its total sum over nodes.

All functionals defined here are derived directly from the measurement-native bounded field $\phi$and depend only on the chosen index adjacency. They are introduced as mathematically well-posed descriptors of variation and energy on a bounded biochemical state space. These quantities do not represent thermodynamic energy, heat exchange, dissipation, or microscopic irreversibility.

Supplemental Note 9 | Site-wise bounded tropical geometry of redox state changes

Let $\phi \in [0,1]^N$denote the vector of site-wise cysteine oxidation occupancies measured for a given condition, where each component corresponds to a single cysteine residue. For two conditions $A$and B (e.g. young and old), define the site-wise change field

$$\Delta = \phi^{(B)} - \phi^{(A)}.$$

For each site i, the initial occupancy $x_{A,i} = \phi_i^{(A)}$defines a starting point in the bounded unit interval, and the pair $(x_{A,i}, \Delta_i)$ uniquely represents the site-wise redox transition between conditions.

Bounded site-wise change space

Because oxidation occupancy is bounded to the unit interval, admissible changes satisfy the hard constraints

$$-x_{A,i} \leq \Delta_i \leq 1 - x_{A,i}.$$

Hence, all site-wise transitions lie within a closed, triangular region of the (x_A, Δ) plane. This feasible region is determined entirely by the bounded state space and does not depend on assumptions about kinetics, dynamics, or regulation.

This construction is measurement-native: no rescaling, normalisation, or projection is applied. The resulting geometry therefore encodes the complete set of physically admissible site-wise redox transitions directly implied by the data.

Symmetric and asymmetric site decomposition

The global redox state of a sample is defined as the arithmetic mean of site-wise occupancies,

$$\bar{\phi} = \frac{1}{N}\sum_{i=1}^{N} \phi_i \,.$$

By linearity, the difference between global means across conditions is given by

$$\bar{\phi}^{(B)} - \bar{\phi}^{(A)} = \frac{1}{N}\sum_{i=1}^{N} \Delta_i \,.$$

Sites with $\Delta_i = 0$ contribute identically to both conditions and therefore cannot influence the global shift. Consequently, any non-zero global change must arise exclusively from sites exhibiting signed change.

This motivates a geometric decomposition of the lattice into:

- a **symmetric subspace**, defined by sites with $| \Delta_i | \leq \varepsilon$, and
- an **asymmetric subspace**, defined by sites with $| \Delta_i | > \varepsilon$,

where ε is a fixed tolerance reflecting measurement precision. This decomposition is exact and parameter-free aside from the explicit tolerance choice.

Headroom-limited action and signed collapse

Although each site formally has one degree of freedom, any realised change is constrained by its available headroom. For a site with initial oxidation x_A, the maximum admissible oxidation is $1 - x_A$, while the maximum admissible reduction is x_A.

Thus, a realised change represents a signed collapse constrained by these limits. For example, a site at $x_A = 0.4$ may increase by at most 0.6 or decrease by at most 0.4; if a decrease occurs, no further reduction is possible without violating the bounded state space. These constraints impose strict geometric limits on site-wise physical action.

Normalised signed redox change

To enable comparison of site-wise changes across different starting occupancies, a normalised signed redox change is defined

$$a_i = \begin{cases} \dfrac{\Delta_i}{1 - x_{A,i}}, & \Delta_i > 0 \\ \dfrac{\Delta_i}{x_{A,i}}, & \Delta_i < 0 \\ 0, & |\Delta_i| \leq \varepsilon, \end{cases}$$

which yields a bounded coordinate $a_i \in [-1,1]$.

This representation preserves directionality while expressing each realised change as a fraction of the site's available redox freedom. Importantly, a is a representation-only quantity: it does not encode dynamics, rates, or energetic costs, but provides a constraint-respecting geometric descriptor of site-wise redox change.

Summary

Together, the bounded change space (x_A, Δ), the symmetric/asymmetric decomposition, and the normalised signed coordinate $a$provide a complete geometric description of site-wise redox state transitions implied by bounded occupancy measurements. These constructions depend only on the unit-interval constraint and arithmetic structure of the data and therefore constitute an exact, assumption-free geometric encoding of site-wise redox reorganisation.
