# Supplementary material for: A tropical geometry for bounded biochemical state spaces
Source: Patterns (N Y). 2026 Jul 9;7(10):101613. doi: 10.1016/j.patter.2026.101613 (PMC13674305; doi:10.1016/j.patter.2026.101613)
Supplement: Supplementary file 2 [file mmc2.pdf]

Article

Patterns

A tropical geometry for bounded biochemical state spaces

Graphical abstract

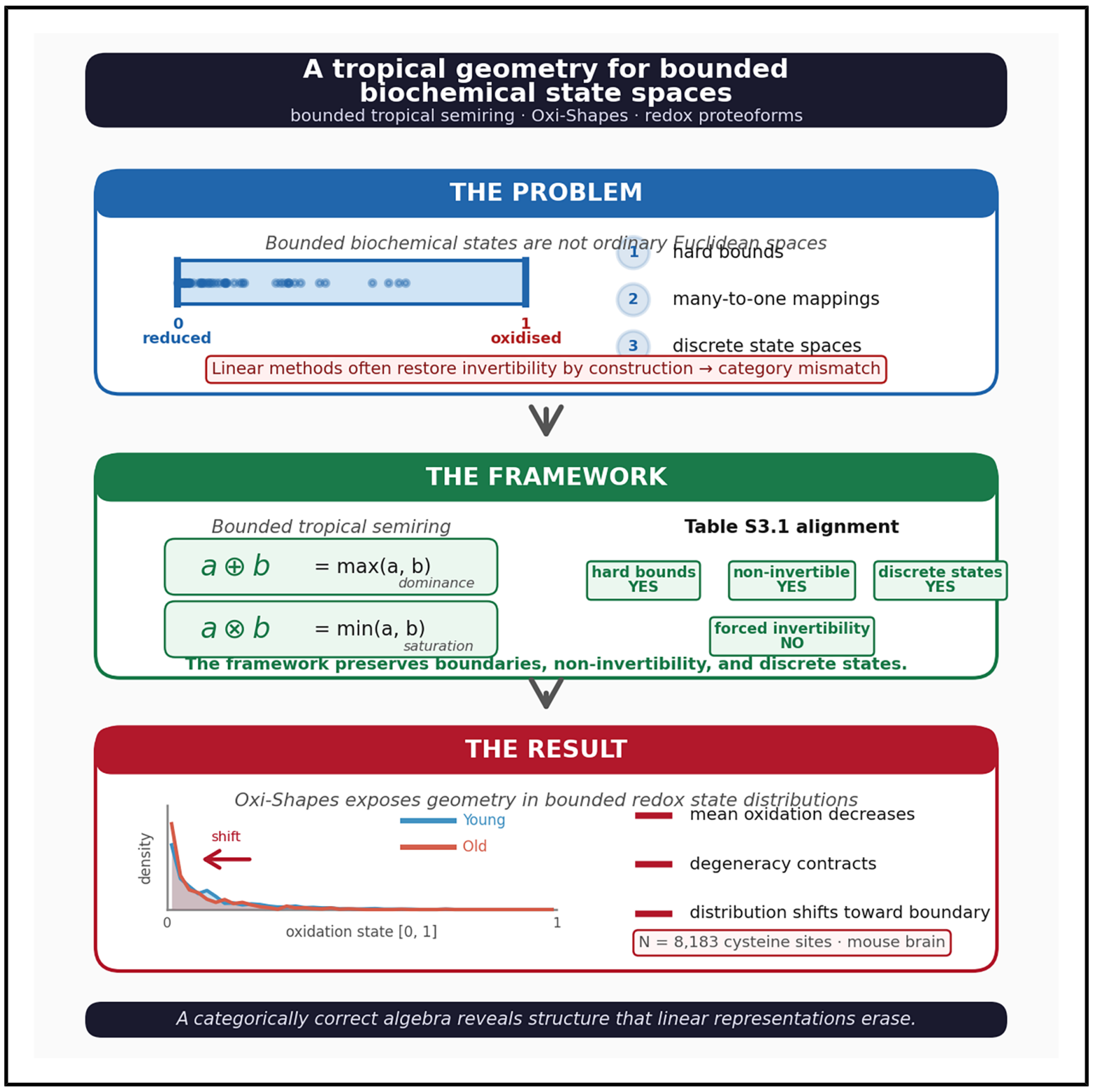

Authors

James N. Cobley

Correspondence

jcobley001@dundee.ac.uk

In brief

Many biological measurements are bounded: a protein site cannot be less than unmodified or more than fully modified. Cobley shows that treating such data with ordinary linear tools can hide biological structure. Using cysteine oxidation as an example, Oxi-Shapes applies tropical geometry to reveal how aging reorganizes the redox proteome despite small average changes, offering a broader framework for analyzing bounded biochemical state spaces.

Highlights

- Bounded biochemical data require boundary-aware algebra
- Oxi-Shapes maps cysteine oxidation as a tropical redox lattice
- Aging contracts accessible cysteine redox configuration space
- Small mean shifts mask large site-wise redox reorganization

Cobley, 2026, Patterns 7, 101613
October 9, 2026 © 2026 The Author. Published by Elsevier Inc.
https://doi.org/10.1016/j.patter.2026.101613

CellPress

Patterns

CellPress
OPEN ACCESS

Article

A tropical geometry for bounded biochemical state spaces

James N. Cobley[1,2,*]

[1]School of Life Sciences, The University of Dundee, Dow Street, Dundee DD1 5EH, Scotland, UK

[2]Lead contact

*Correspondence: jcobley001@dundee.ac.uk

https://doi.org/10.1016/j.patter.2026.101613

THE BIGGER PICTURE Biological measurements are often treated as ordinary numbers, even when the underlying biology does not behave like an ordinary line. Many biochemical states are bounded: a protein site can be unmodified or modified, reduced or oxidized, but it cannot move beyond these physical limits. Such boundaries are not statistical inconveniences. They change what a measurement means, how histories collapse, and what future changes remain possible.

This paper argues that the mathematics used to analyze biochemical data must match the structure of the biological object being measured. For bounded state spaces, conventional linear and Euclidean representations can obscure information because they assume properties the data do not possess, including unbounded translation and global reversibility. A small numerical change can therefore hide a large change in the underlying configuration of the system.

Using cysteine oxidation as a concrete example, this work introduces Oxi-Shapes, a tropical geometric representation of redox proteomic data. Rather than treating oxidation values as unconstrained linear quantities, Oxi-Shapes represents them as bounded occupancies on a discrete lattice of invariant cysteine sites. Applied to aging mouse brain data, the framework reveals that modest changes in mean oxidation correspond to a substantial contraction of the accessible redox configuration space and extensive site-wise reorganization.

The broader message is simple: biological data should be analyzed in an algebra that respects the limits, asymmetries, and irreversibility of the state space. This principle extends beyond cysteine redox biology to other bounded biochemical measurements, including post-translational modification occupancies and proteoform-resolved states.

SUMMARY

Many biochemical measurements define state spaces that are bounded, absorbing, and generically noninvertible yet they are routinely analyzed using categorically mismatched linear and Euclidean frameworks. This categorical mismatch can irretrievably obscure biological structure. This work formalizes the minimal algebraic properties imposed by bounded biochemical state spaces, demonstrating that a bounded tropical semiring provides a natural category match by replacing additive linear structure with order-based, piecewise-linear operations that encode dominance, saturation, and path-dependence without contradiction. To instantiate this framework, measurement-native tropical geometric analysis was applied to cysteine redox proteomic data (Oxi-Shapes). Oxi-Shapes represents cysteine oxidation occupancies as a bounded scalar field over a discrete lattice of invariant residue identities, enabling analysis of both ensemble-level organization and site-wise admissible transitions. As demonstrated on empirical cysteine redox proteomic data, categorically correct algebraic structures exposed geometric features that remained inaccessible under unbounded linear representations.

This is an open access article under the CC BY license (http://creativecommons.org/licenses/by/4.0/).

INTRODUCTION

Categorically correct data representation and analysis require matching the algebra to the object of study in biology.[1] Categorical mismatches arise when the algebraic framework applied to a dataset assumes structural properties—global invertibility, unbounded translation, or symmetric state spaces—that the data do not and cannot possess by formal construction. If the algebra is categorically mismatched by construction, then normalization, transformation, or modeling cannot recover the lost structure.[2] This loss arises from algebraic incompatibility rather than scale or noise. Hence, mismatched algebraic representations can produce concomitant losses of biological meaning, hampering discovery-driven analysis.[3]

Protein post-translational modifications (PTMs), such as phosphorylation, ubiquitination, and oxidation, define bounded biochemical state spaces.[4] Chemically reversible cysteine oxidation, for instance, regulates protein structure and function and can now be quantified across thousands of sites via redox proteomics.[5–15] These measurements report ensemble occupancies bounded to the unit interval [0,1] defined over discrete, invariant residue identities. Hard boundaries, absorbing states, and many-to-one ensemble mappings are intrinsic features of these state spaces.

Despite these properties, bounded PTM data are routinely analyzed using linear and Euclidean frameworks that assume global invertibility, symmetry, and translation invariance.[16,17] Operations such as averaging, projection, and variance maximization preserve these assumptions by formal construction, even when applied to bounded data. However, in state spaces with absorbing boundaries and generically irreversible ensemble dynamics, equal-magnitude changes do not encode equal biological meaning, trajectories cannot be globally inverted, and intermediate states constrain future possibilities. These violations are structural rather than statistical, rendering linear algebra categorically mismatched to bounded biochemical objects.

To address this mismatch, this work formalizes the minimal algebraic structure required to represent bounded biochemical state spaces, thereby demonstrating that a bounded tropical semiring provides a natural category match. In this framework, order-based, piecewise-linear operations replace additive linear structure, boundaries are intrinsic rather than imposed, and noninvertibility and path-dependence arise without contradiction. A measurement-native geometric instantiation of this bounded tropical structure (Oxi-Shapes) is applied to high-dimensional redox proteomic data. Using invariant cysteine sites as a discrete lattice and bounded oxidation occupancies as a scalar field, categorically correct representation enables the definition of geometric objects describing both ensemble-level organization and site-wise admissible transitions. This establishes a foundation for analyzing bounded biochemical state spaces without imposing algebraic assumptions that contradict their structure.

RESULTS

Overview

This work formalizes the structure of bounded biochemical state spaces and identifies the minimal algebraic properties required for categorically correct representation. Hard boundaries, absorbing states, and many-to-one ensemble mappings impose structural constraints that are incompatible with linear and Euclidean frameworks, independent of normalization or preprocessing. Rather than introducing a new analytical procedure, this work formally establishes these constraints and provides geometric interpretations that motivate their algebraic resolution via a bounded tropical framework. Formal definitions, propositions, and proofs are provided in Notes S1–S9.

Building on this fixed mathematical representation, this work constructs a measurement-native geometric realization of high-dimensional redox proteomic data. The algebraic structure of the bounded tropical state space is instantiated as a discrete lattice with a bounded scalar field (Oxi-Shapes), independent of any specific dataset, and subsequently applied to empirical redox proteomic data.[18]

The results are organized around geometric objects intrinsic to bounded biochemical state spaces rather than around analytical procedures. These objects fall into two complementary classes. Global geometric objects describe ensemble-level organization of the redox proteome, including order-disorder, entropy, and energy-like structure. Local geometric objects describe site-wise admissible transitions within the bounded interval, capturing capacity, feasibility, and directionality of redox change at individual residues. For each object, this work establishes what can and cannot be inferred under many-to-one observation and then demonstrates these consequences empirically. Each result therefore reflects a necessary consequence of categorically correct representation rather than an outcome of optimization, modeling, or algorithmic choice.

Structure of bounded biochemical state spaces

Many biochemical measurements are naturally described as state variables constrained to a finite interval (Figure 1). As a representative case, cysteine redox states are commonly quantified as an occupancy bounded between the fully reduced (0) and fully oxidized (1) limits.[19] These bounds are physically immutable constraints: a cysteine molecule or population cannot be more reduced than 0 or more oxidized than 1.[20] These scale-invariant bounds apply to both the protein-level object—the cysteine proteoform[21–23]—and its component parts—each individual cysteine site. Resultantly, the underlying state space is closed and bounded, and algebraic operations that assume unbounded translation are immediately incompatible.

The boundaries of this space act as absorbing states.[24] A large fraction of cysteines reside at or near the fully reduced boundary,[18] and many distinct biochemical trajectories collapse onto the same observed state. Once this collapse occurs, information about prior history is irretrievably lost at the level of observation—the act of measurement. Consequently, the mapping from biochemical trajectories to measured states is generically many-to-one, rendering the state space noninvertible. For example, for a cysteine proteoform with three cysteines considered in redox binary, the 000 state could arise from any of the 100, 010, and 001 biochemical states (3:1 mapping).[20]

In bounded state spaces, equal-magnitude changes do not encode equal biological meaning. For example, a shift from 0.0 to 0.1 represents the emergence of oxidation from an absorbing boundary, whereas a shift from 0.5 to 0.6 reflects redistribution within an already oxidized ensemble. Conceptually, this

CellPress
OPEN ACCESS

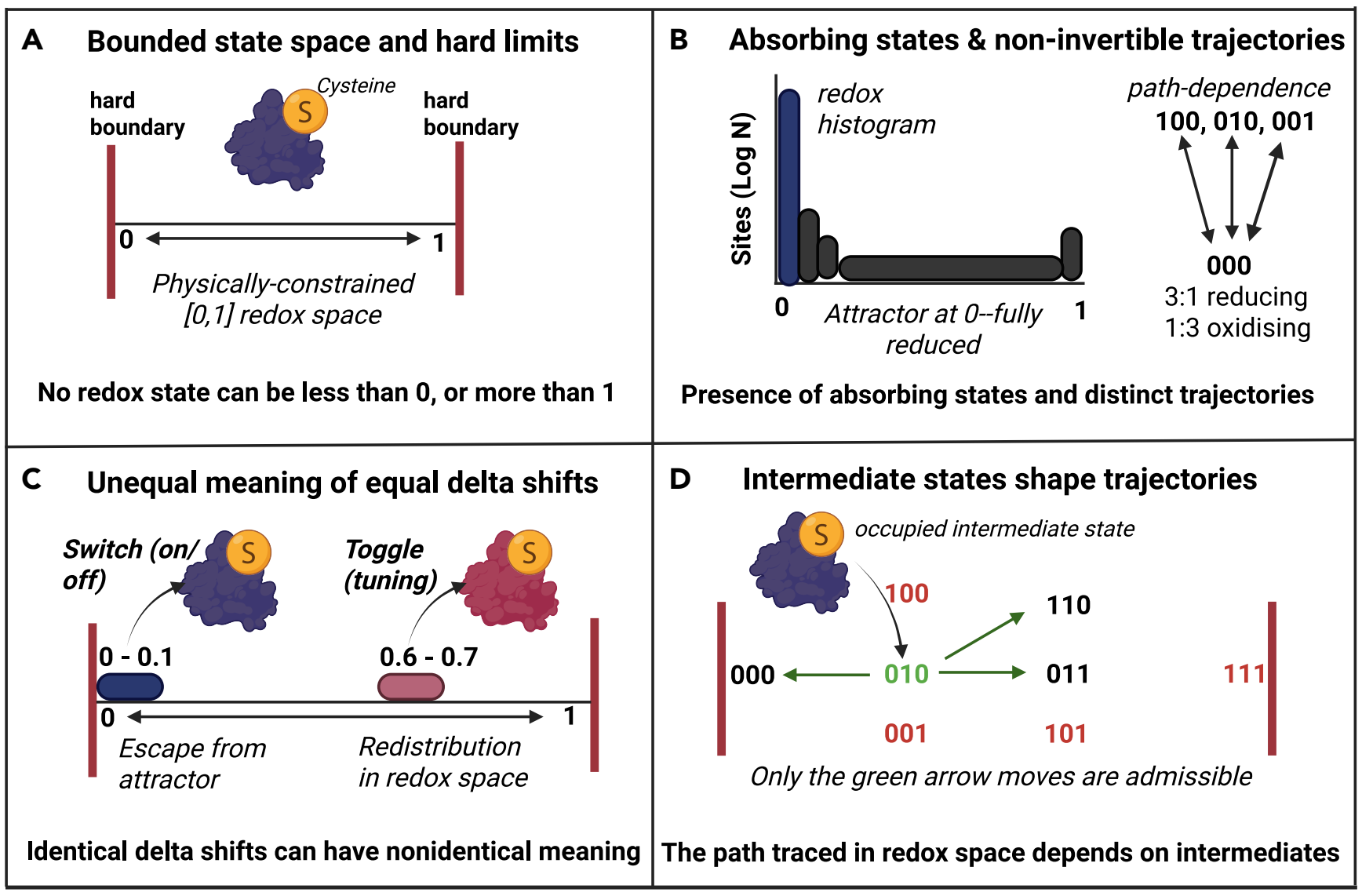

Figure 1. Structural properties of bounded biochemical state spaces illustrated by cysteine oxidation

(A) Cysteine redox states are constrained to a physically immutable, closed interval [0,1], corresponding to fully reduced (0) and fully oxidized (1) ensembles. No biochemical or observational state can exist outside these hard boundaries.

(B) The bounded state space contains absorbing states, illustrated by the strong empirical bias toward the fully reduced boundary. Distinct biochemical trajectories collapse onto common observed states, producing a many-to-one mapping and rendering the state space non-invertible.

(C) Equal-magnitude changes in redox occupancy encode unequal biological meaning depending on position within the bounded space. Small shifts near the reduced boundary correspond to switch-like activation (e.g., escape from an attractor), whereas identical shifts within the interior reflect tuning or redistribution within an already oxidized ensemble.

(D) Intermediate states constrain admissible trajectories in discrete proteoform space. Only transitions consistent with occupied intermediates are allowed, introducing path-dependence even when individual redox reactions are chemically reversible.

represents the difference between emergent "switch" and "toggle" behavior, respectively.[25] Although numerically identical, these changes differ fundamentally in their physical interpretation and in the constraints that they impose on subsequent state evolution.[20]

While individual redox reactions may be chemically reversible,[26] ensemble-level redox dynamics are generically physically irreversible due to nonnegative entropy production[27–29] (e.g., via heat dissipation). Transitions that reach or approach absorbing boundaries reduce the accessible state space for future directional evolution (e.g., toward the fully reduced state), introducing path-dependence into the system.[20] As a result, the sequence in which biochemical transitions occur matters: intermediate states constrain subsequent possibilities even when the net chemical change is nominally reversible.

Together, hard boundaries, absorbing states, and physical irreversibility define the essential structure of bounded biochemical state spaces.[30] These properties are intrinsic to the object and must be respected by any algebraic representation intended to analyze such data. Formal definitions and proofs of the consequences of these properties are provided (Note S1).

Algebraic incompatibility of linear representations

Linear and Euclidean representations assume global invertibility, symmetry, and translation invariance of the underlying state space.[16] These assumptions are preserved under common operations such as normalization, averaging, and dimensionality reduction, which rescale or reorient data without altering its algebraic structure. As formally demonstrated (Note S2), the properties of bounded biochemical state spaces are categorically incompatible with the assumptions of linear algebra.

Minimal algebraic requirements for bounded state spaces

Any algebraic framework suitable for representing bounded biochemical state spaces must satisfy a minimal set of requirements imposed by the object itself. The algebra must do the following:

(1) respect hard bounds without relying on unconstrained translation,
(2) accommodate absorbing states and many-to-one mappings without assuming global invertibility,
(3) represent irreversible, path-dependent transitions without assuming symmetry or commutativity, and
(4) remain well-defined on discrete or combinatorial state spaces,[31] such as proteoform ensembles.[32–35]

These requirements follow directly from the structural properties established above and therefore constrain any admissible algebraic representation.

While this work does not claim it is the only representation, tropical algebra provides a natural realization of these requirements by replacing additive linear structure with order-based, piecewise-linear operations. In tropical geometry, bounds are intrinsic rather than imposed, absorbing states arise naturally through dominance, and irreversible transitions are represented without contradiction. Tropical operations remain well-defined on discrete state spaces, allowing direct representation of proteoform-level objects without invoking infinitesimal neighborhoods or smooth flows. Alternative boundary-aware approaches—such as logit transformation, beta regression, or compositional methods—handle boundedness by mapping observations into an unbounded space prior to analysis, thereby restoring invertibility by construction and concealing the noninvertibility that is intrinsic to the biological object (Note S3).

Tropical representation of bounded biochemical state spaces

To make the algebraic representation explicit,[36–38] this work encodes cysteine redox states as bounded occupancies $x \in [0,1]$,

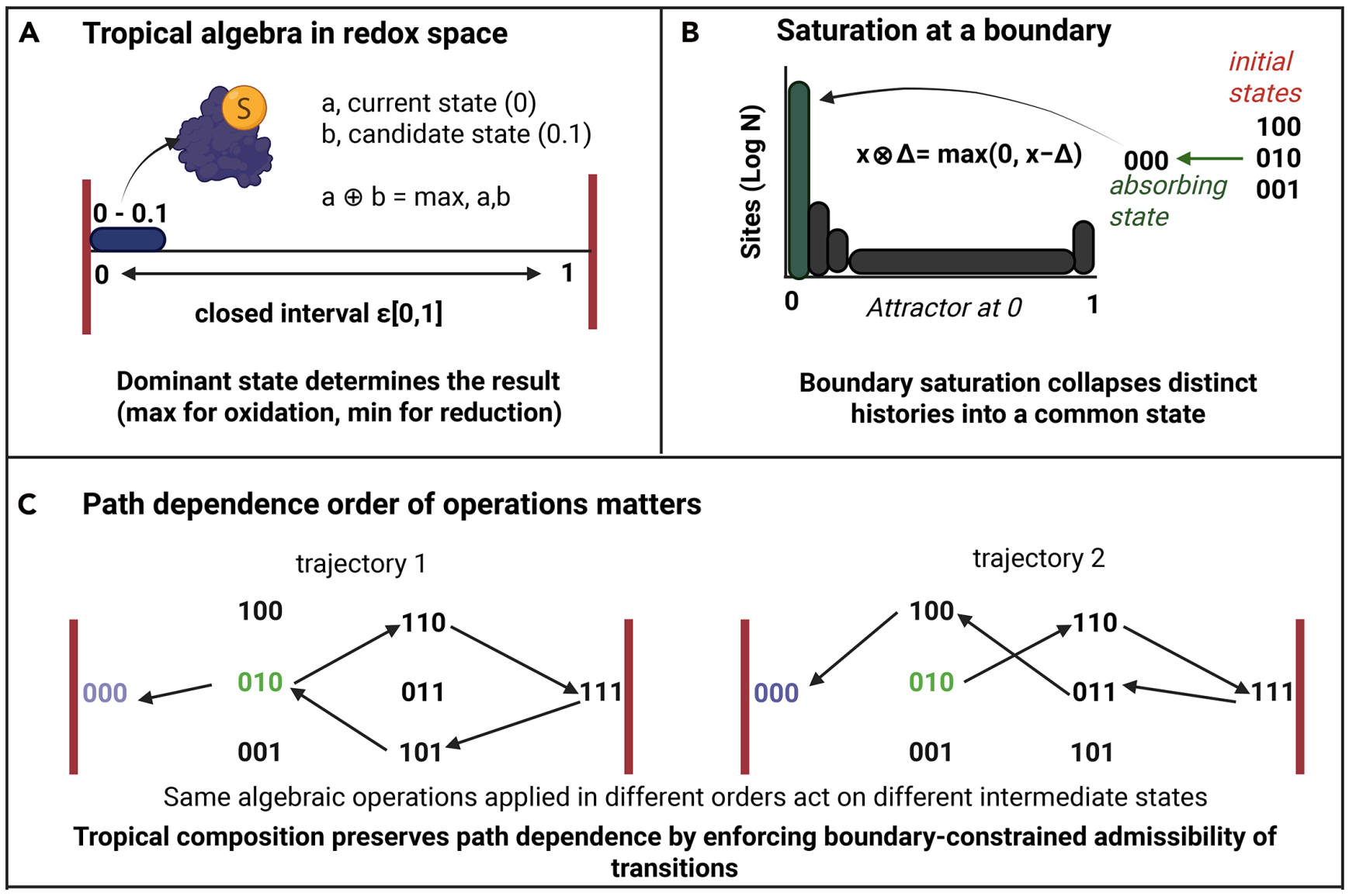

Figure 2. Tropical algebra provides a categorically correct representation of bounded redox state evolution

(A) Tropical addition acting on residue-level redox occupancies $x \in [0,1]$ selects the dominant state via order-based comparison ($a \oplus b = max(a,b)$), enforcing a closed, physically constrained interval. Oxidation and reduction are represented without invoking unbounded translation.

(B) Boundary saturation at the fully reduced state ($x = 0$) acts as an absorbing state. Distinct initial proteoform configurations collapse onto a common observed state under boundary-constrained composition, rendering the mapping from trajectories to observations many to one and non-invertible.

(C) Path-dependence emerges from boundary-constrained admissibility of transitions. The same algebraic operations applied in different orders act on different intermediate states, yielding distinct trajectories even when the net operations are identical. Tropical composition preserves path-dependence by enforcing state-dependent constraints on allowable transitions.

where $x = 0$ denotes a fully reduced ensemble and $x = 1$ denotes a fully oxidized ensemble. Changes in redox state therefore correspond to ordered transitions within a closed interval—a bounded tropical semiring—rather than additive displacements on an unbounded axis (Figure 2).

In a tropical representation, linear addition is replaced by order-based operations acting directly on a bounded biochemical observable. For a residue-level occupancy $x \in [0,1]$, tropical addition is defined as

$$a \oplus b = \max(a, b),$$

which encodes dominance and biological saturation. Sequential biochemical transitions are composed via

$$a \otimes b = \min(1, a + b),$$

ensuring closure on the bounded state space. Here, x denotes the current biochemical state, while b represents a residue-level redox increment; state evolution is defined by the tropical action of such increments on the current occupancy. Under this formulation, oxidation events increase occupancy toward the upper boundary, reduction events decrease occupancy toward the lower boundary, and operations that leave the state unchanged under $\oplus$ encode biological saturation rather than the absence of activity.

Noninvertibility arises naturally as a structural consequence of the bounded, absorbing state space. Multiple distinct biochemical trajectories may converge to the same boundary value, irreversibly collapsing information about prior history at the level of observation. Once this collapse occurs, no global inverse mapping can exist, even though individual redox reactions may be chemically reversible. Tropical geometry makes this loss of invertibility explicit rather than concealing it behind assumptions of smoothness or reversibility.

It is important to distinguish between algebraic operations and state evolution under their composition. Individual tropical operations are typically commutative (e.g., $a \oplus b = b \oplus a$). However, the induced evolution of biochemical states is noncommutative in bounded, irreversible spaces. The order in which redox events—and more generically PTMs—occur matters because intermediate states constrain subsequent possibilities. An oxidation followed by reduction need not yield the same outcome as reduction followed by oxidation if one sequence encounters a boundary or dissipative loss before the other.

A similar distinction applies to associativity. While tropical operations themselves are associative, the evolution of biochemical states under grouped sequences of transitions becomes path-dependent due to boundary constraints. Grouping transitions differently can lead to distinct intermediate states, and these intermediate states affect the admissible set of future transitions. As a result, biochemical state evolution is inherently path-dependent, even when individual transformations are well-defined.

Hard boundaries therefore play an active and explicit role in tropical representations. Once a state reaches a boundary, further operations in the same direction leave the state unchanged, reflecting biological saturation rather than mathematical stagnation. In this sense, boundaries act as absorbing states: they terminate certain classes of trajectories and collapse distinct histories into a common endpoint, analogous to extremal regions in tropical geometry where dominance becomes degenerate.

As a concrete illustration, consider a single cysteine residue with initial occupancy $x = 0.92$. Under a linear representation, an oxidation increment of +0.1 would formally yield $x = 1.02$, a state that is biologically and observationally impossible. In the tropical representation, the same transition is composed as

$$x \otimes 0.1 = \min(1, 0.92 + 0.1) = 1,$$

explicitly encoding saturation at the hard boundary. Any subsequent oxidation leaves the state unchanged under $\oplus$, while reduction transitions act relative to this saturated state. Distinct oxidation histories that reach the boundary therefore collapse onto the same observed value, making the loss of invertibility explicit.

Formal definitions of the bounded tropical state space, proofs of noninvertibility and path-dependence, and extensions to discrete proteoform ensembles are provided (Notes S4 and S5).

CellPress
OPEN ACCESS

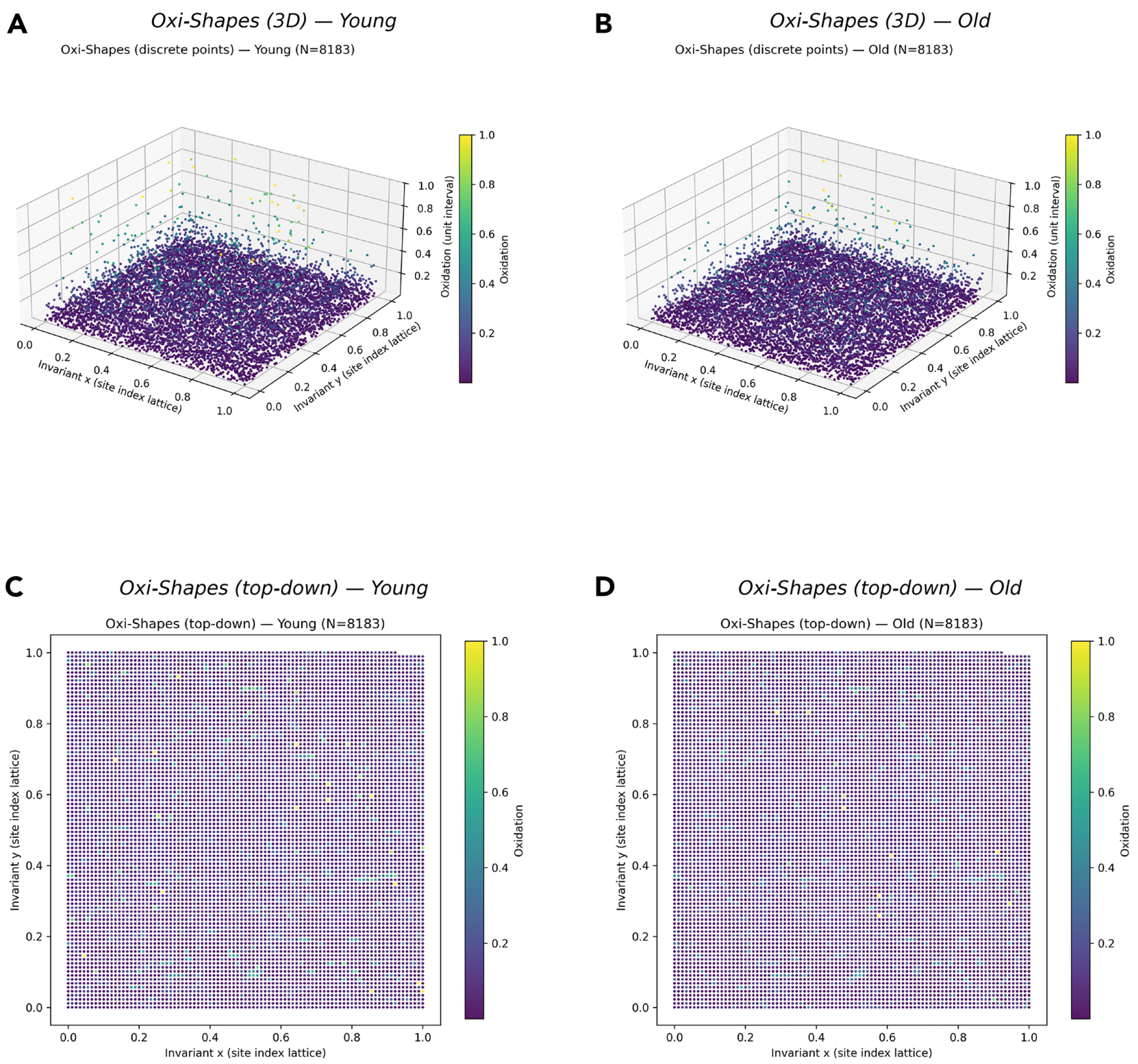

Figure 3. The cysteine redox proteome as a discrete tropical lattice

Each point corresponds to an invariant cysteine site positioned on a discrete lattice index set. Color and height indicate the measured oxidation occupancy bounded to the unit interval [0,1]. Top-down (B and D) and three-dimensional (A and C) views are shown for the same object. Young (A and B) and old (C and D) mouse brain datasets are displayed as example instantiations of the lattice populated by experimental measurements.

The cysteine redox proteome as a high-dimensional discrete tropical lattice

Having established the algebraic structure of bounded biochemical state spaces, this work now constructs a measurement-native geometric realization of this bounded tropical framework in redox proteomic data. Cysteine oxidation is quantified as bounded occupancies in the [0,1] state space. To represent these data as high-dimensional objects, each cysteine residue is assigned a unique and invariant position in a discrete lattice index set, providing a fixed correspondence between measured sites and lattice elements across conditions. Measured oxidation values are then realized as bounded scalar values along a third (oxidation) axis over this lattice (Note S6), yielding a discrete tropical representation in which structure is induced directly by the measured biochemical state rather than by imposed relational or embedding assumptions.

In Oxi-Shapes, each cysteine site therefore carries an intrinsic state variable—its oxidation occupancy—that is mutable across conditions but conserved at the level of information: site identity is invariant, and each measurement assigns a well-defined bounded value whenever the site is observed. This property enables direct information-theoretic[39] interpretation of the measured redox states (i.e., non-probabilistic) without logarithmic transforms, binning procedures, or assumptions of an unbounded state space.

To make this representation concrete, an example instantiation of the cysteine redox proteome—computed in young and old mouse brain[18]—in which each invariant lattice element corresponds to a measured cysteine site and the bounded oxidation occupancy is shown as a scalar height (Figure 3). The brain was selected due to its sensitivity to redox dysregulation.[40–42] This example serves solely to illustrate the measurement-native lattice object populated by experimental data; no relational structure or geometric operators are introduced at this stage. As a measurement-native structure, the lattice object does not encode biological embeddings, such as grouping of indexes by pathway terms.[43–45]

Internal redox entropy: Order-disorder in the bounded cysteine state space

In Oxi-Shapes, internal redox entropy defines a volume-based measure of order-disorder on the discrete cysteine lattice (Note S7). By construction, each site contributes exactly one bounded scalar value in [0,1], and the total volume of the object is given by the arithmetic mean across sites. This quantity corresponds to the average uplift of the lattice along the bounded oxidation axis, providing a direct geometric measure of how evenly reduced and oxidized states are distributed across the admissible state space (Figure 3).

Maximum internal order corresponds to every site occupying either the 0 (reducing bias) or 1 (oxidizing bias) state, which geometrically maps to a perfectly flat lattice at 0 (zero volume) or 1 (maximal volume). In a bounded binary state space, maximum internal disorder occurs at 0.5 occupancy, corresponding to the region of greatest configurational degeneracy. While no dynamical behavior is assumed or measured here, this point represents the most disordered macrostate in the admissible configuration space, from which no additional disorder can be gained without altering the system constraints.

Internal redox entropy is not a measure of thermodynamic entropy and makes no direct claim about heat exchange, dissipation, or microscopic irreversibility.[27,28,46] The measured configurations instead reflect these properties insofar as they arise from an open biological system, but they are not themselves measurements of external entropy production. Rather, internal redox entropy represents a geometric projection of configurational structure defined on a bounded biochemical state space at a fixed observational snapshot. Consistent with a tropical representation, this construction is inherently mesoscopic and non-invertible: multiple biochemical histories may map to the same observed configuration,[47] while dynamical irreversibility remains outside the scope of the present analysis.

Applying this framework to shared cysteine sites quantified in young and old mouse brain (N = 8,183 sites) provides a proof-of-principle instantiation of internal redox entropy in a biological system rather than a test of a statistical hypothesis. Within this framework, aging was associated with a decrease in internal redox entropy of the cysteine proteome, reflected as a reduction in mean oxidation from young to old brain (young = 0.0889; old = 0.0814; $\Delta = -0.0074$).

Although the absolute change in mean oxidation is numerically small, its interpretation must be made relative to the bounded state space. The young proteome begins at 0.0889, meaning the maximal possible shift toward a fully reduced state is itself only 0.0889. The observed decrease therefore represents a substantial fraction of the available reducing headroom. Both young and old proteomes occupy a highly ordered regime near the saturating 0 boundary, where additional reductions in mean correspond to progressively stronger constraints on admissible configurations.

Consistent with this geometry, internal redox entropy was numerically identical to the arithmetic mean in both conditions, confirming that the volume measure introduces no additional transformations beyond those inherent to the bounded representation. This identity is constitutive of the framework. In an unbounded linear setting, the arithmetic mean carries no entropic interpretation—it is a descriptive statistic. In a bounded state space, the same quantity axiomatically indexes the system's state along the configurational degeneracy curve, as every possible mean value corresponds to a specific and computable number of admissible configurations. The entropic interpretation therefore arises directly from the boundedness of the representation, without additional assumptions, transforms, or models. Internal redox entropy names this interpretation.

When mapped onto the combinatorial structure of the binary state space,[31] the observed shift in internal redox entropy corresponded to a pronounced reduction in configurational degeneracy. Despite similar mean oxidation, the young and old proteomes differed by approximately 63 orders of magnitude in the number of admissible configurations consistent with their respective means (Figure 4). Importantly, this structure is constitutively absent from linear representations of the same data: a framework that does not encode the bounded state space has no language for configurational degeneracy, regardless of the sophistication of the analysis applied.

The discrete lattice visualization captures the specific realized configuration in each condition, while the degeneracy curve quantifies the size of the surrounding admissible configuration space indexed by the same mean. Together, these results demonstrate that aging of the mouse brain is associated with a measurable contraction of the accessible redox configuration space, even when changes in mean oxidation appear modest. Internal redox entropy therefore provides a geometrically grounded descriptor that distinguishes realized redox organization from the broader space of mathematically admissible alternatives.

Deriving an energy functional from the bounded redox lattice scalar field

In Oxi-Shapes, the primary measurement object is a bounded scalar field $\phi \in [0,1]^N$ defined on a discrete index lattice. Volume-based order-disorder metrics are therefore definable without imposing any neighborhood structure (Notes S7 and S8). However, any energy functional that depends on spatial variation—that penalizes or rewards changes in ϕ across the lattice—requires an explicit relational operator.

This work therefore introduces a minimal, index-defined adjacency on the lattice and uses the associated graph Laplacian to derive curvature-like and energy-like functionals from the same measurement-native field, enabling high-dimensional geometric analysis of the lattice object. It does not encode physical interaction networks, spatial proximity, reaction pathways, or temporal transitions between cysteine residues. Neighbor relations are introduced solely to enable geometric comparison of bounded state values and carry no biological interpretation beyond this formal role. Importantly, these constructions do not claim thermodynamic energy measurement; they define mathematically well-posed functionals on a bounded biochemical state space that enable consistent comparisons across conditions (Note S9).

Applying a minimal index-defined adjacency enabled the computation of curvature- and energy-like functionals directly from the measured redox lattice without altering the underlying scalar field. The resulting graph Laplacian curvature exhibited zero mean in both young and old proteomes, as required by construction (Figure 5A), but showed a systematic reduction in dispersion with age (young SD = 0.437; old SD = 0.376). This narrowing of curvature fluctuations indicates a reduction in local geometric variation across the lattice, consistent with a loss of configurational heterogeneity in the aged proteome despite similar global ordering. Importantly, curvature here reflects discrete second-order variation of the measured field and does not imply spatial diffusion, interaction, or dynamics.

From this relational structure, the Dirichlet energy, which quantifies quadratic variation of oxidation values across the lattice, was defined. Dirichlet energy was substantially lower in old relative to young brains (ΔE_D = −41.3 total; −26% per-node mean), indicating reduced spatial roughness of the redox field with age (Figure 5B). Hence, the topology shifted toward a "flatter" state, indicative of less volume (Figures 3 and 4).

Likewise, a Morse-type potential with a ground state at ϕ = 0 yielded lower mean and total energy in the old proteome (Δ mean = −0.012; Δ sum = −98.6), reflecting a global shift toward lower energetic displacement from the reducing ground state (Figure 5C). Put differently, the Morse energy reflects the volume deflating toward the ground state—0—indicating a contraction toward an absorbing 0 state.

Without involving thermodynamics or kinetics, these results demonstrate that aging is associated with a coordinated reduction in curvature and energy-like variation across the bounded biochemical state space.

Site-wise bounded tropical geometry of redox state changes

Next, the geometry of site-wise redox state changes within the bounded cysteine state space was examined (Note S9). For each cysteine residue, the measured oxidation in the initial condition defines a starting point $x_A \in [0,1]$, while the change between conditions is given by $\Delta = x_B - x_A$. Each site is therefore represented as a point (x_A,Δ) in a constrained two-dimensional

CellPress
OPEN ACCESS

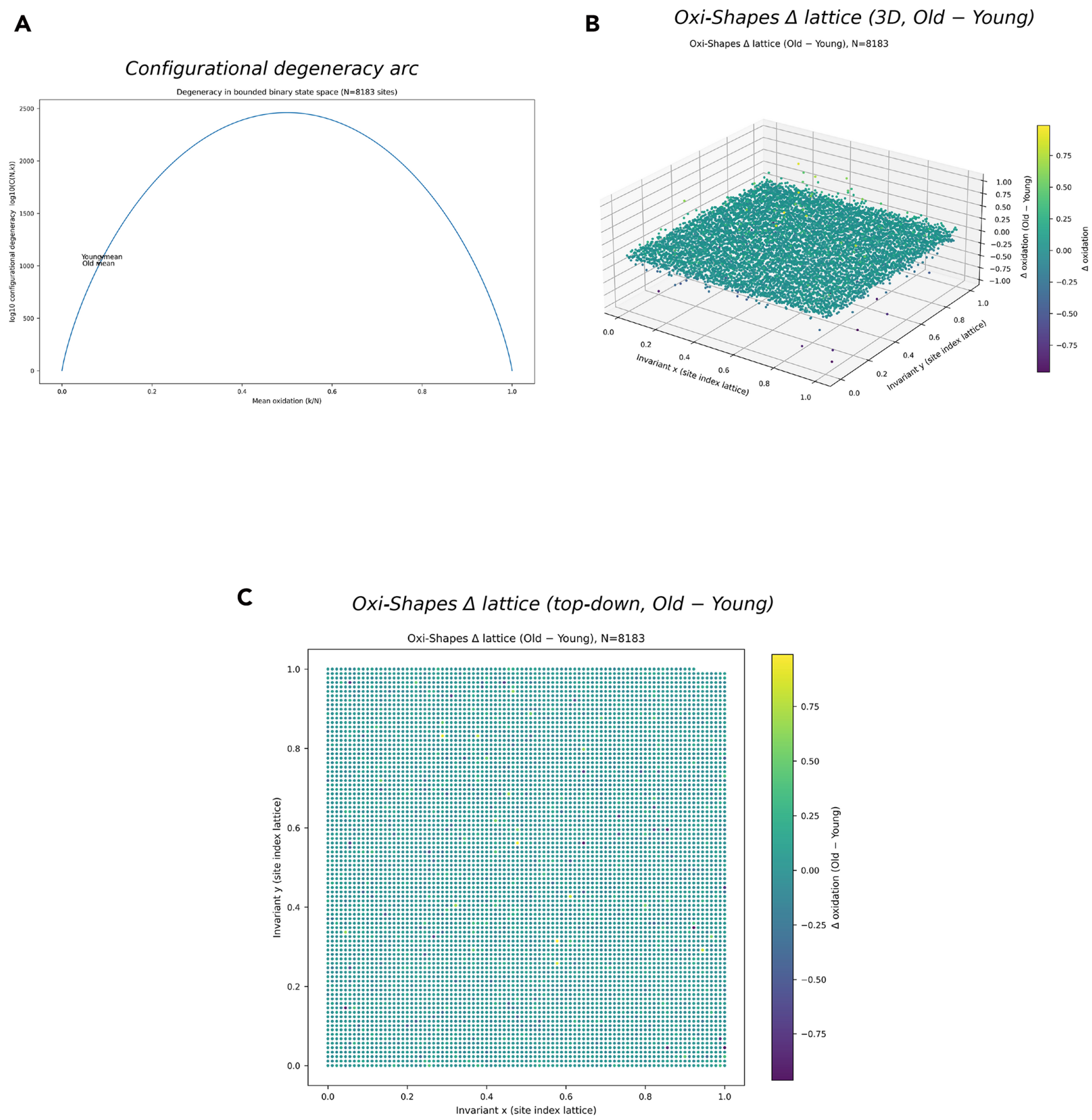

Figure 4. Internal redox entropy and configurational degeneracy in the aging mouse brain

(A) Configurational degeneracy in a bounded binary state space for N = 8,183 cysteine sites. The number of admissible configurations consistent with a given mean oxidation (k/N) is given by the binomial coefficient Ω = C (N, k), shown here on a $\log_{10}$ scale. The resulting inverted-U curve reflects maximal degeneracy at 0.5 occupancy and minimal degeneracy at the boundaries. Vertical markers indicate the observed mean oxidation values for young and old mouse brain, both of which lie deep within the low-occupancy, highly ordered regime.

(B) Three-dimensional Oxi-Shapes Δ-lattice showing site-wise changes in oxidation between old and young mouse brain (old − young). Each point corresponds to a single cysteine site positioned on the invariant lattice; color and height encode the signed change in oxidation within the bounded interval [−1, 1]. Despite the modest shift in the global mean, the lattice reveals a structured redistribution of oxidation states across sites.

(C) Top-down view of the same Δ-lattice, emphasizing the spatial distribution of redox changes without imposing adjacency or continuity.

Together, (B and C) visualize the realized configuration corresponding to the observed change in internal redox entropy, complementing the abstract degeneracy structure shown in (A).

space whose admissible region is fully determined by the boundedness of oxidation occupancy.

This representation is exact and measurement native. No rescaling, normalization, or linear projection is applied, and the feasible region $-x_A \leq \Delta \leq 1 - x_A$ follows directly from the unit interval constraint. The geometry of this site-wise change space therefore encodes the full set of physically admissible redox transitions without imposing assumptions about dynamics, kinetics, or biological coupling between residues.[48]

Because the global redox state is defined as the arithmetic mean of site-wise occupancies, any difference between global means must arise exclusively from sites that change state. Sites with $\Delta \approx 0$ contribute identically to both conditions and therefore cannot influence the global shift. Consequently, the observed change in global redox organization is necessarily encoded entirely in the subset of sites exhibiting non-zero signed change. This property follows directly from the linearity of the mean and is independent of any assumptions about mechanism or regulation.

This observation enables a natural geometric decomposition of the lattice into symmetric and asymmetric components. Sites with $|\Delta|$ below a specified tolerance define a symmetric subspace that preserves the global redox state across conditions, while sites with signed changes define an asymmetric subspace that drives the observed shift. The balance between these components provides a site-resolved encoding of global redox symmetry breaking without invoking statistical modeling or parameter fitting.

Site-wise redox change is strictly bounded by the starting occupancy of each site. For a site with initial oxidation x_A, admissible changes satisfy $-x_A \leq \Delta \leq 1 - x_A$. This constraint implies that although each site formally has one degree of freedom, any realized change represents a signed collapse constrained by its available headroom. For example, a site at $x_A = 0.4$ may increase by at most 0.6 or decrease by at most 0.4. If a decrease occurs, no further reduction is possible without violating the bounded state space. These hard geometric limits impose strict constraints on physical action at the level of individual residues and make explicit how identical numerical changes can carry fundamentally different physical meaning depending on starting state.

To enable comparison of site-wise changes across different starting occupancies, a normalized signed redox change that expresses each realized Δ as a fraction of the site's available redox freedom was defined. For a site with initial oxidation x_A, the normalized change is defined as $a = \Delta/(1 - x_A)$ for oxidation and $a = \Delta/x_A$ for reduction, yielding a bounded coordinate $a \in [-1,1]$. This representation preserves directionality while removing dependence on absolute headroom, providing a constraint-respecting, representation-only descriptor of site-wise redox change.

Applying this site-wise bounded representation to shared cysteine sites quantified in young and old mouse brain

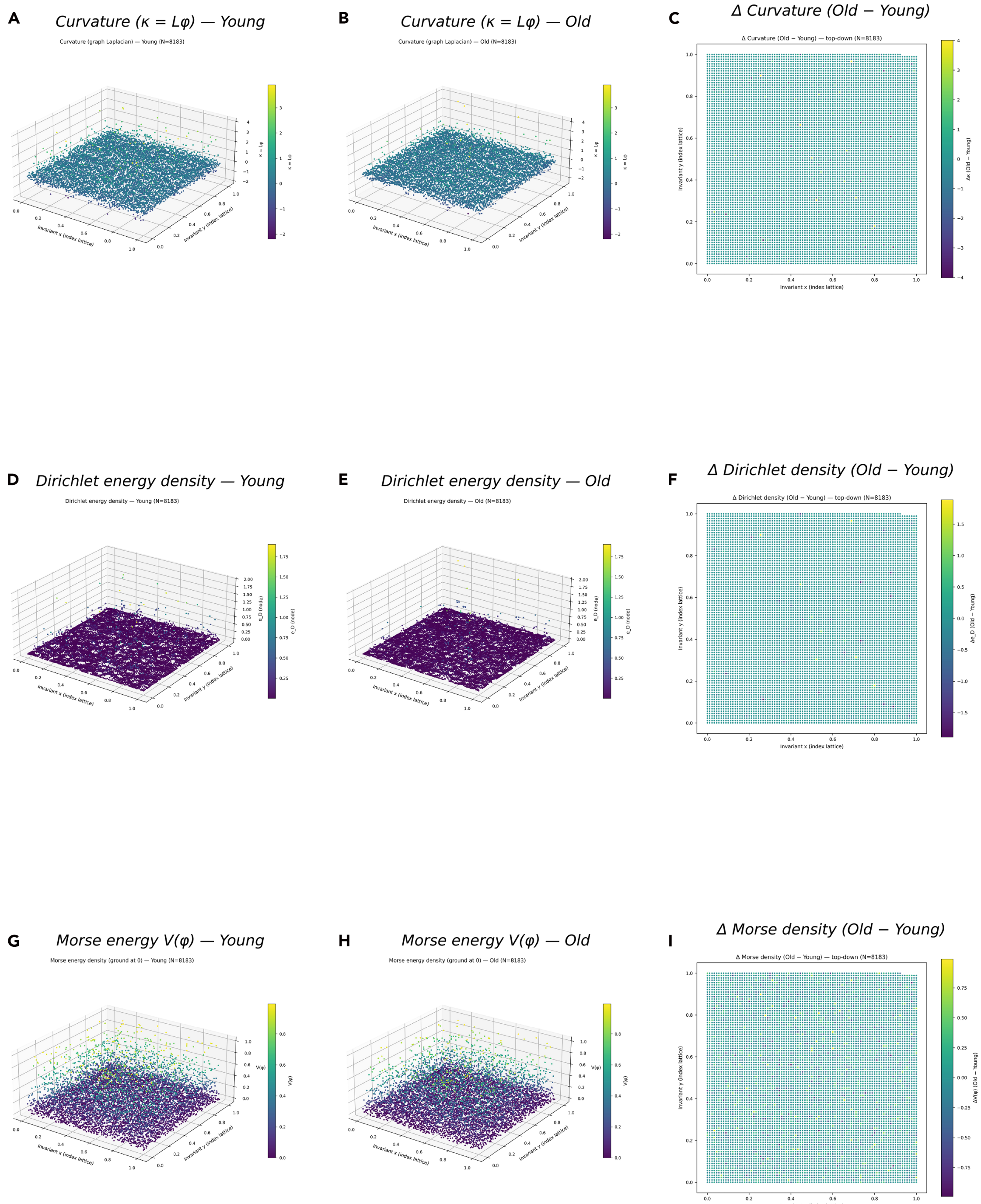

Figure 5. Curvature and energy functionals derived from the bounded redox lattice scalar field

(A–C) Graph Laplacian curvature ($\kappa = L\phi$) computed on the discrete index lattice for young (A and old (B mouse brain proteomes (N = 8,183 shared cysteine sites), visualized as three-dimensional Oxi-Shapes with lattice indices on the horizontal axes and curvature on the vertical axis. The corresponding top-down difference map (C) shows $\Delta\kappa = \kappa\_old - \kappa\_young$. By construction, curvature has zero mean and captures second-order variation of the bounded scalar field.

(D–F) Dirichlet energy functional density per node, quantifying quadratic variation of oxidation values across adjacent lattice indices, shown for young (D) and old (E) conditions, with the top-down difference map (F) indicating a global reduction in Dirichlet energy with age.

(G–I) Morse energy functional density with a ground state at $\phi = 0$, shown for young (G) and old (H) proteomes, and corresponding top-down difference map (I). This measurement-native functional assigns increasing energy cost to displacement from the fully reduced state while remaining defined entirely on the bounded scalar field.

CellPress
OPEN ACCESS

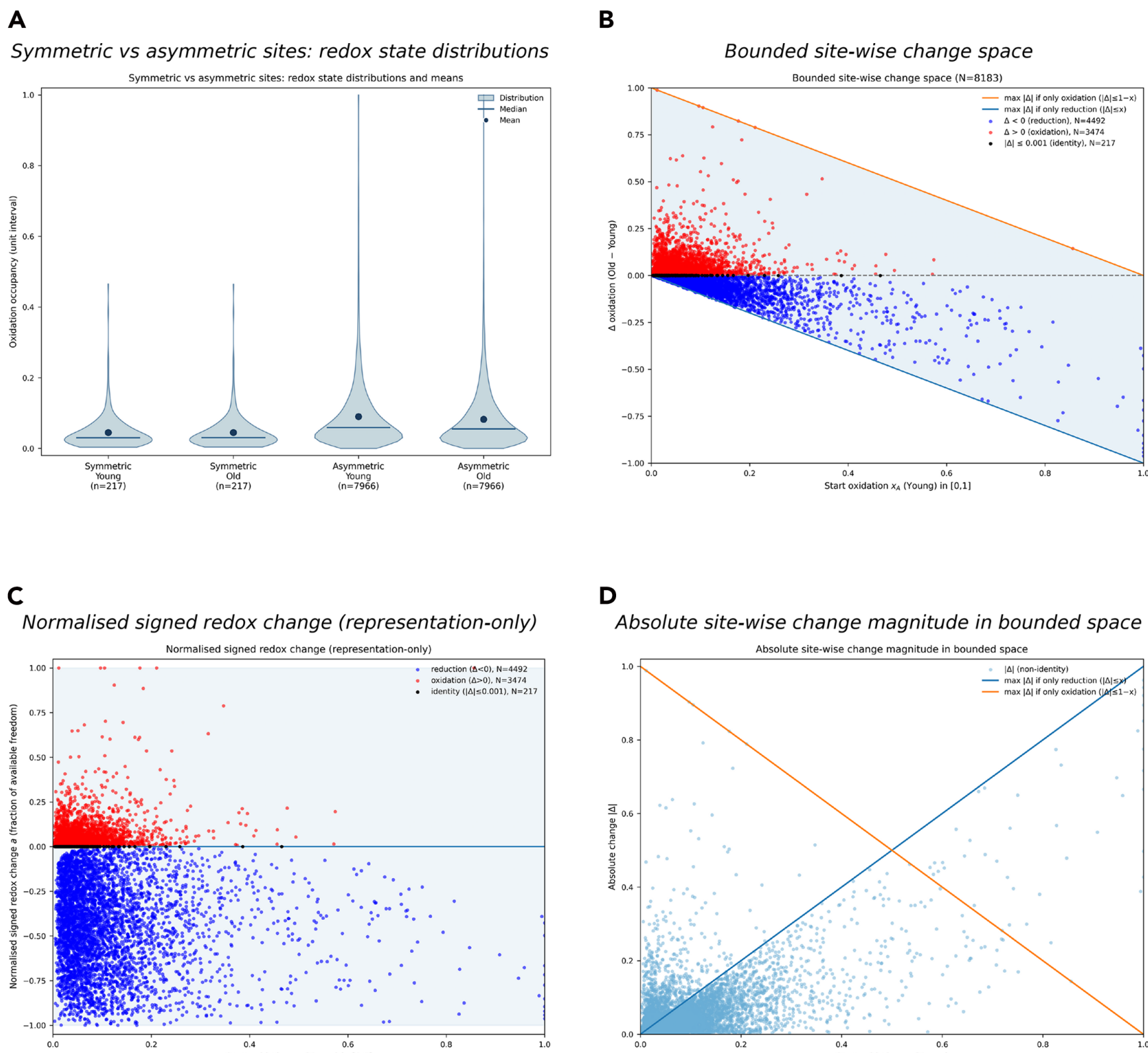

Figure 6. Site-wise bounded tropical geometry of cysteine redox state changes

(A) Violin plots showing the distribution of oxidation occupancies for symmetric sites ($|\Delta| \leq 0.001$) and asymmetric sites ($|\Delta| > 0.001$) in young and old mouse brains. Points and horizontal bars indicate means. Symmetric sites contribute identically to both conditions and therefore do not influence the global redox shift, whereas asymmetric sites encode the entirety of the observed change.

(B) Exact site-wise change space represented as starting oxidation x_A (young) vs. signed change $\Delta = x_B - x_A$. The admissible region is strictly bounded by $-x_A \leq \Delta \leq 1 - x_A$, shown by the triangular envelope. Points are colored by direction of change (reduction, oxidation, or identity), illustrating the hard geometric constraints imposed by the bounded state space.

(C) Normalized signed redox change a, defined as the signed fraction of available headroom used by each site. This representation collapses site-wise changes onto a common scale $[-1,1]$ while preserving directionality, making explicit how identical absolute changes correspond to different fractions of available freedom depending on starting state.

(D) Absolute site-wise change magnitude $|\Delta|$ as a function of starting oxidation, shown together with the maximal admissible change envelopes for pure reduction ($|\Delta| \leq x_A$) and pure oxidation ($|\Delta| \leq 1 - x_A$). This image highlights how large absolute changes are geometrically restricted near the boundaries of the state space.

(N = 8,183), the mean oxidation decreased modestly with age (young = 0.0889; old = 0.0814; Δ = $-$0.00742). Consistent with the linearity of the mean, this global shift was driven exclusively by sites exhibiting non-zero signed change. Using a tolerance of $|\Delta| \leq 0.001$ to define identity, 217 sites (2.7%) were classified as symmetric, while 7,966 sites (97.3%) formed the asymmetric subspace encoding the entire global redox difference (Figures 6A and 6B). Reconstructing the global mean change from asymmetric sites alone reproduced the observed Δ to numerical precision.

Site-wise changes occupied the full admissible region of the bounded change space, with Δ spanning from $-$0.963 to +0.988 depending on starting oxidation. When expressed as a normalized signed redox change (Figures 6C and 6D), many sites exhibited values approaching $|a| \approx 1$, indicating near-complete utilization of available redox freedom despite modest absolute changes. Across asymmetric sites, the distribution of a was skewed toward negative values (mean a = $-$0.223), consistent with the observed global shift toward a more reduced proteome. These results demonstrate that the bounded tropical geometry yields a faithful, constraint-respecting decomposition of global redox change into site-resolved contributions without invoking statistical modeling or dynamical assumptions.

Despite exhibiting similar global mean oxidation, the young and old redox lattices are almost completely divergent in their site-wise compositions. The small net shift in the mean is produced by a large redistribution of individual cysteine states, with thousands of sites undergoing signed, bounded transitions rather than remaining invariant. Notably, a substantial subset of sites exhausts nearly all available redox freedom, collapsing toward the fully reduced (0) absorbing boundary, revealing extensive geometric reorganization beneath an apparently modest global change.

DISCUSSION

Motivated by the goal of representing biochemical measurements using a categorically correct algebraic formalism, this work formalized the structure of bounded biochemical state spaces and constructed a measurement-native tropical geometric instantiation in redox proteomic data. Hard boundaries, absorbing states, and many-to-one ensemble mappings are intrinsic properties of such objects. These properties impose algebraic constraints that are incompatible with linear and Euclidean representations, independent of normalization, transformation, or modeling choices. As a result, this work introduced important concepts that are defined in plain terms in Table 1.

The incompatibility arises from categorical mismatch rather than from noise, scale, or insufficient data. In bounded state spaces, trajectories are generically noninvertible, equal-magnitude numerical changes do not encode equal biological meaning, and intermediate states constrain future evolution. Linear frameworks, which assume global invertibility, symmetry, and translation invariance, therefore conflate distinct biochemical histories and obscure the geometry of the underlying state space.

Within this formally defined structure, Oxi-Shapes provides a concrete geometric realization operating both at the level of the

Table 1. Plain-language summary of selected key terms

Term	Plain-language meaning	Analogy
Absorbing boundary	a physical limit that a biochemical state cannot cross and from which it cannot escape by further change in the same direction; once reached, the system is trapped there	a ball rolling into a valley—once it settles at the bottom, pushing it further downhill is impossible
Configurational degeneracy	the number of distinct ways the same average oxidation level could be distributed across all cysteine sites; a high-degeneracy state has many equivalent arrangements; a low-degeneracy state has very few	the same hand total in cards can be dealt in many different combinations—degeneracy counts how many
Lattice curvature	a measure of how sharply oxidation values differ between neighboring sites; high curvature means the redox landscape is rough and heterogeneous; low curvature means it is flat and uniform	the difference between a jagged mountain range and a gently rolling plain, viewed from above
Energy-like functional	a mathematical quantity, analogous to physical energy but not measuring it directly, that quantifies how far the system's configuration is from a reference state (here, fully reduced); lower values indicate the proteome is collectively closer to the reduced ground state	a stretched elastic band stores more potential energy the further it is pulled from rest—the functional measures the equivalent displacement in biochemical state space

global lattice and at the level of site-wise admissible transitions. Computational instantiation in the Oxi-Mouse dataset demonstrated the consequences of correct representation. Aging was associated with a decrease in internal redox entropy of the cysteine proteome, corresponding to a substantial contraction in the configurational degeneracy available to the global lattice. This shift was accompanied by reduced curvature dispersion and lower Dirichlet and Morse-energy-like functionals, indicating a flattening of the lattice topology toward the absorbing reduced boundary.

Mathematically, these global shifts arose from extensive reorganization at the site level. Thousands of cysteine residues altered occupancy, and many sites approached or exhausted their available redox headroom. Despite only modest changes in the arithmetic mean oxidation, the underlying configuration of the bounded state space changed profoundly. Similar means therefore corresponded to dramatically different combinatorial structures, consistent with the high degeneracy of low-occupancy regimes in bounded spaces.

The present framework does not reproduce or replace the landmark analysis of the OxiMouse[18] study. Rather, it reveals a geometric and combinatorial structure in the same data that is constitutively absent from a linear representation—not overlooked by the original analysis but inexpressible within it. The tropical framework reveals that the observed decrease corresponds to a contraction of approximately 63 orders of magnitude in configurational degeneracy—a consequence of boundary proximity with no equivalent in fold-change analysis. The tropical framework additionally quantified that 97.3% of sites are asymmetric and that many exhausted nearly all available redox headroom—a boundary-aware quantity that fold change cannot encode. The Morse energy functional revealed a coordinated contraction toward the absorbing reduced boundary that is invisible to site-level or network-level analysis but emerges directly from the measurement-native representation. Together, these results demonstrate that categorically correct representation exposes geometric structure that conventional analysis cannot access, even when applied to the same dataset.

Importantly, the geometric quantities defined here do not measure thermodynamic entropy or physical energy. Rather, they provide well-posed functionals on a bounded biochemical state space that expose order-disorder structure and admissible transitions without imposing dynamical or mechanistic assumptions. They reveal the structural shadow of biochemical action on the proteome while respecting the epistemic limits of snapshot measurement. Distinct biochemical trajectories can collapse onto the same observed configuration, and dynamics cannot be uniquely reconstructed from ensemble occupancies alone.

Although cysteine redox states served as a concrete instantiation, the formal arguments extend beyond redox biology. Many biochemical measurements define bounded state spaces with absorbing boundaries and generically irreversible ensemble behavior—including stoichiometric occupancies of other PTMs (e.g., phosphorylation) and protein group abundances where physical limits on copies per cell define hard boundaries that, once made explicit via normalization against estimated maximum copy number, satisfy the same qualifying criteria. The tropical representation introduced here is therefore applicable wherever categorical mismatches between algebra and object arise, and where such mismatches exist, the framework additionally motivates new representational questions—for example, how protein abundance measurements should be scaled to recover a physically valid bounded space—that conventional linear analysis does not generate. This work does not claim that tropical algebra is the only possible resolution, nor that all biological systems

require tropical representations. Rather, it establishes a general principle: categorically correct analysis requires that the algebra respect the structural properties of the object under study. When this condition is violated, no amount of post-hoc transformation can recover the lost structure.

From a theoretical perspective, the measurement-native construction of internal redox entropy yields a precise prediction: configurational degeneracy is maximal at 0.5 occupancy, where a site has equal capacity for oxidation and reduction. A logical consequence is that measurement variability should peak at this point—not as noise to be minimized but as a structural feature of sites with maximum bidirectional freedom. Many partially oxidized sites may therefore be intrinsically unstable outside the reduced ground state of the system, and the near-universal redistribution of sites observed with age is consistent with this geometry. More fundamentally, the formal proof of the bounded state space places a constraint on the application of chaos theory[49] to cysteine redox dynamics. Classical chaos concerns the indefinite divergence of trajectories from nearby initial conditions, but a cysteine redox butterfly is formally caged: two trajectories cannot diverge beyond the hard limits of the bounded interval, and sensitivity to initial conditions is itself bounded by the available headroom at the starting occupancy. Extending dynamical and chaos-theoretic frameworks to bounded biochemical state spaces therefore requires explicit mathematical adjustment to respect these structural constraints—a direction this work opens rather than closes.

Ensuring algebra-object compatibility is therefore a prerequisite for meaningful interpretation of bounded biological data. By making the structural constraints of bounded biochemical state spaces explicit and providing a concrete geometric instantiation, this work establishes a foundation for representation and analysis that is aligned with the intrinsic properties of the biological object.

METHODS

Redox proteomics dataset

Redox proteomics data were obtained from the Oxi-Mouse dataset,[18] in which cysteine oxidation was quantified as the percentage of occupancy of individual cysteine residues across biological conditions. For the analyses shown here, oxidation measurements from young and old mouse brain samples were used as representative instantiations of the redox state space.

Oxidation values were expressed as percentages or fractions depending on source and converted to unit interval representation [0,1] prior to analysis. All downstream analyses were performed on this bounded state space.

Preprocessing and site selection

To ensure that comparisons were performed on invariant molecular identities, analyses were restricted to cysteine sites measured in both conditions under consideration. Sites with missing oxidation values in either condition were excluded. Each cysteine site was assigned a stable and deterministic identifier derived from available metadata (e.g., protein accession, residue position, and peptide sequence). When no explicit site identifier was available, the row index was used as a fallback. This ensured that each site retained a unique and invariant identity across conditions and analyses. Oxidation values were converted to the unit interval [0,1].

Discrete lattice construction

To construct the Oxi-Shapes object, each invariant cysteine site was embedded in a discrete two-dimensional lattice index space. Site identifiers were first sorted deterministically, and lattice coordinates were assigned sequentially on a near-square grid. The resulting coordinates were normalized to [0,1] for visual consistency across datasets of different sizes.

This lattice embedding is index-based only and serves to provide a discrete base manifold for representing bounded state measurements. It does not encode biological adjacency, interaction, similarity, or spatial proximity between cysteine residues.

For each condition, the measured oxidation occupancy of each cysteine site was represented as a bounded scalar value assigned to its corresponding lattice position. The resulting object is a high-dimensional discrete lattice with a scalar field defined over it, fully determined by experimental measurements. The object therefore reflects only invariant site identity and bounded biochemical state, consistent with the formal construction (Notes S1–S9).

Oxi-Shapes were visualized using both top-down and three-dimensional scatterplots. In the three-dimensional view, lattice indices define the horizontal axes and oxidation occupancy defines the vertical axis. In the top-down view, oxidation occupancy is encoded by color. All visualizations were rendered as discrete point clouds without interpolation, triangulation, or smoothing, to avoid implicitly imposing continuity or neighborhood structure. Color scales and axis limits were shared across conditions to ensure direct comparability.

Computation of internal redox entropy and configurational degeneracy

Internal redox entropy was computed directly from the bounded cysteine oxidation measurements. For a given condition, internal redox entropy was defined as the arithmetic mean of site-wise oxidation occupancies across all invariant cysteine sites retained for analysis. Because each site contributes exactly one bounded scalar value in [0,1], this quantity is numerically identical to the total lattice volume scaled by the number of sites, consistent with the formal definition of the Oxi-Shapes object (Notes S1–S9).

To verify numerical consistency, internal redox entropy was explicitly compared to the arithmetic mean oxidation for each condition, and equivalence was confirmed to machine precision. The difference in internal redox entropy between conditions was reported as the difference in mean oxidation.

Configurational degeneracy associated with a given internal redox entropy value was computed under a bounded binary-state approximation. For a system of N cysteine sites and a measured mean oxidation V, the effective number of oxidized sites was defined as $k = \text{round}(N \times V)$. The number of distinct configurations consistent with this mean was then given by the binomial coefficient

$$\Omega(N,k) = \binom{N}{k}.$$

Degeneracy values were reported on a logarithmic scale due to their large magnitude.

To visualize the structure of the admissible configuration space, degeneracy was computed across the full bounded range of mean oxidation values from 0 to 1 in fixed increments, yielding a characteristic inverted-U curve with maximal degeneracy at 0.5 occupancy. Observed mean oxidation values for each biological condition were mapped onto this curve to contextualize the realized configurations within the surrounding admissible configuration space.

This analysis is deterministic and proof of principle in nature. No statistical hypothesis testing was performed. Internal redox entropy and configurational degeneracy were computed solely to characterize the geometric and combinatorial structure of the bounded cysteine state space defined by experimental data.

Computation of lattice curvature and derived energy functionals

To derive energy-like descriptors from the bounded redox lattice, a minimal relational structure was introduced on the discrete index grid. A 4-neighbor adjacency (von Neumann neighborhood) was imposed on the two-dimensional lattice used for Oxi-Shapes visualization, connecting each lattice site to its immediate horizontal and vertical neighbors where present. This adjacency is index defined only and does not encode biological interaction networks, spatial proximity, reaction pathways, or temporal transitions between cysteine residues.

The resulting adjacency structure defines a graph $G = (V,E)$ with $|V| = N$ nodes corresponding to invariant cysteine sites and edges connecting neighboring lattice indices. From this graph, the unnormalized graph Laplacian was constructed as

$$L = D - A,$$

where A is the adjacency matrix and D is the diagonal degree matrix.

Laplacian curvature was computed by applying the graph Laplacian to the bounded oxidation field,

$$\kappa = L\phi,$$

yielding a discrete second-difference operator that quantifies local variation of oxidation values relative to neighboring lattice indices. Curvature values were summarized by their mean and standard deviation and visualized as both three-dimensional lattice scatterplots and top-down difference maps between conditions.

A quadratic variation energy functional (Dirichlet energy) was computed as

$$E_D(\phi) = \frac{1}{2}\sum_{(i,j)\in E}\left(\phi_i - \phi_j\right)^2 = \frac{1}{2}\phi^\top L\phi.$$

Total Dirichlet energy was reported for each condition, along with a per-node energy density obtained by normalizing by the number of sites. This quantity measures the aggregate spatial variation of oxidation values across the lattice and depends only on relative differences between adjacent lattice indices.

To encode an explicit ground-state bias at the fully reduced state ($\phi = 0$), a bounded Morse-type potential was applied independently to each lattice site,

$$V(\phi_i) = \left(1 - e^{-a\phi_i}\right)^2,$$

with parameter $a = 6.0$. Morse energy was summarized as both the mean and total energy across all sites. This functional depends only on the absolute oxidation value at each site and does not require neighborhood interactions.

Construction of the bounded site-wise change space

Site-wise redox state changes were analyzed using a bounded tropical geometric representation derived directly from experimental measurements. For each invariant cysteine site measured in both conditions, the oxidation occupancy in the initial condition (young) was denoted as

$$x_A \in [0,1],$$

and the change between conditions was defined as

$$\Delta = x_B - x_A,$$

where x_B is the oxidation occupancy in the comparison condition (old).

Each cysteine site was therefore represented as a point (x_A,Δ) in a two-dimensional bounded change space. Because oxidation occupancy is constrained to the unit interval, admissible changes are strictly bounded by

$$-x_A \le \Delta \le 1 - x_A.$$

This feasible region arises directly from the boundedness of the underlying biochemical measurement and was not imposed algorithmically. Sites violating these bounds were explicitly checked for, and none were observed beyond numerical tolerance.

This representation is measurement native and exact. No rescaling, transformation, regression, or dimensionality reduction was applied. The geometry of the site-wise change space therefore encodes the complete set of physically admissible redox transitions consistent with the experimental data.

Classification of symmetric and asymmetric sites

To decompose the site-wise change space, cysteine residues were classified according to the magnitude of their signed change. Sites with

$$|\Delta| \le \varepsilon$$

were classified as symmetric (identity preserving), where $\varepsilon = 10^{-3}$ corresponds to a tolerance of 0.1% oxidation occupancy. Sites with $|\Delta|>\varepsilon$ were classified as asymmetric and represent residues contributing to differences between conditions.

This classification follows directly from the linearity of the arithmetic mean: sites with $\Delta \approx 0$ contribute identically to both conditions and therefore cannot influence the global mean oxidation, whereas sites with non-zero signed change necessarily encode all observed differences between global states. No statistical modeling or hypothesis testing was applied to define these classes.

Normalized signed redox change (fraction of available freedom)

To enable comparison of site-wise changes across different starting occupancies, a normalized signed redox change

CellPress
OPEN ACCESS

coordinate was defined. For each asymmetric site, the realized change Δ was expressed as a fraction of the site's available redox headroom,

$$a = \begin{cases} \dfrac{\Delta}{1 - x_A}, & \text{if } \Delta > 0(\text{oxidation}) \\ \dfrac{\Delta}{x_A}, & \text{if } \Delta < 0(\text{reduction}) \\ 0, & \text{if} |\Delta| \leq \varepsilon. \end{cases}$$

This yields a bounded, dimensionless coordinate $a \in [-1,1]$, where the sign encodes directionality and the magnitude encodes the fraction of available redox state space utilized by the observed change. Values of $a = \pm 1$ correspond to sites that fully exhaust their admissible redox headroom in the observed direction.

This normalized signed redox change is a representation-only descriptor. It preserves directionality while removing dependence on absolute starting occupancy and does not assume kinetic, temporal, or mechanistic equivalence between sites.

Ranking of site-wise action magnitude

To provide an ordering of site-wise redox changes independent of absolute scale, the absolute normalized change $|a|$ was ranked among asymmetric sites using a dense rank normalized to the unit interval. This delta rank provides a representation-only measure of relative action magnitude within the bounded change space. Rank values were used exclusively for visual encoding and descriptive reporting and were not interpreted as probabilities or statistical significance measures.

Visualization of site-wise redox geometry

Four complementary visualizations were generated.

(1) Bounded site-wise change space: scatterplot of (x_A,Δ) with explicit rendering of the admissible region $-x_A \leq \Delta \leq 1 - x_A$, colored by directionality (oxidation, reduction, and identity).
(2) Absolute change magnitude: plot of $|\Delta|$ as a function of x_A, overlaid with the theoretical upper bounds imposed by oxidation and reduction headroom.
(3) Normalized signed redox change: scatterplot of (x_A,a), with point size optionally encoding delta rank.
(4) Symmetric vs. asymmetric distributions: violin plots comparing oxidation occupancy distributions in young and old conditions for symmetric and asymmetric site classes, with arithmetic means overlaid.

All plots were rendered directly from discrete data points without interpolation, smoothing, or kernel density estimation beyond standard violin plot construction. Axis limits and color encodings were fixed across conditions to ensure direct comparability.

Deterministic decomposition of global mean change

As a consistency check, the contribution of asymmetric sites to the global mean oxidation difference was computed explicitly. Because symmetric sites satisfy $\Delta \approx 0$ by construction, the global mean change can be reconstructed as the mean Δ among asymmetric sites weighted by their fraction of the total site count. Agreement with the directly computed global mean difference confirms that all observed changes in global redox state arise exclusively from asymmetric site-wise contributions. This decomposition is deterministic and follows directly from the linearity of the arithmetic mean.

RESOURCE AVAILABILITY

Lead contact

Requests for further information and resources should be directed to and will be fulfilled by the lead contact, James N. Cobley (jcobley001@dundee.ac.uk).

Materials availability

No new materials were created within this study.

Data and code availability

- The cysteine redox proteomic data are available from https://oximouse.hms.harvard.edu/.
- The original code is available on GitHub (https://github.com/JamesCobley/Oxi-Shapes/tree/main) and archived on DOI https://doi.org/10.5281/zenodo.18411791.[50]

ACKNOWLEDGMENTS

The author thanks Prof. Angus I. Lamond (University of Dundee) and all of the members of the Lamond lab for constructive scientific discussions. The support of an MCR grant (MR/Y013891/1) is gratefully acknowledged. Figures 1 and 2 were created using BioRender and exported with a publication license.

AUTHOR CONTRIBUTIONS

J.N.C. conceived and wrote the paper, performed the analysis, and produced the figures.

DECLARATION OF INTERESTS

The author declares no competing interests.

DECLARATION OF GENERATIVE AI AND AI-ASSISTED TECHNOLOGIES IN THE WRITING PROCESS

During the preparation of this work, the author used ChatGPT (OpenAI) as an AI-assisted tool to support language editing, structural refinement, and clarification of conceptual arguments. All scientific content, theoretical development, interpretations, and conclusions were conceived by the author. The author reviewed, edited, and verified all text generated with AI assistance and takes full responsibility for the content of the published article.

SUPPLEMENTAL INFORMATION

Supplemental information can be found online at https://doi.org/10.1016/j.patter.2026.101613.

Received: January 22, 2026
Revised: May 4, 2026
Accepted: June 12, 2026
Published: July 9, 2026

REFERENCES

1. Spivak, D. (1978). Category Theory for the Sciences (MIT Press).
2. Leek, J.T., and Peng, R.D. (2015). Reproducible research can still be wrong: Adopting a prevention approach. Proc. Natl. Acad. Sci. *112*, 1645–1646. https://doi.org/10.1073/pnas.1421412111.

3. Brenner, S. (2010). Sequences and consequences. Philos. Trans. R. Soc. Lond. B Biol. Sci. *365*, 207–212. https://doi.org/10.1098/rstb.2009.0221.
4. Kitamura, N., and Galligan, J.J. (2023). A global view of the human post-translational modification landscape. Biochem. J. *480*, 1241–1265. https://doi.org/10.1042/bcj20220251.
5. D'Autréaux, B., and Toledano, M.B. (2007). ROS as signalling molecules: mechanisms that generate specificity in ROS homeostasis. Nat Rev Mol Cell Bio *8*, 813–824. https://doi.org/10.1038/nrm2256.
6. Lennicke, C., and Cochemé, H.M. (2021). Redox metabolism: ROS as specific molecular regulators of cell signaling and function. Mol. Cell *81*, 3691–3707. https://doi.org/10.1016/j.molcel.2021.08.018.
7. Holmström, K.M., and Finkel, T. (2014). Cellular mechanisms and physiological consequences of redox-dependent signalling. Nat Rev Mol Cell Bio *15*, 411–421. https://doi.org/10.1038/nrm3801.
8. Sies, H., Belousov, V.V., Chandel, N.S., Davies, M.J., Jones, D.P., Mann, G.E., Murphy, M.P., Yamamoto, M., and Winterbourn, C. (2022). Defining roles of specific reactive oxygen species (ROS) in cell biology and physiology. Nat Rev Mol Cell Bio *23*, 499–515. https://doi.org/10.1038/s41580-022-00456-z.
9. Sies, H., Mailloux, R.J., and Jakob, U. (2024). Fundamentals of redox regulation in biology. Nat. Rev. Mol. Cell Biol. *25*, 701–719. https://doi.org/10.1038/s41580-024-00730-2.
10. Sies, H., and Jones, D.P. (2020). Reactive oxygen species (ROS) as pleiotropic physiological signalling agents. Nat Rev Mol Cell Bio *21*, 363–383. https://doi.org/10.1038/s41580-020-0230-3.
11. Winterbourn, C.C. (2008). Reconciling the chemistry and biology of reactive oxygen species. Nat. Chem. Biol. *4*, 278–286. https://doi.org/10.1038/nchembio.85.
12. Day, N.J., Kelly, S.S., Lui, L.Y., Mansfield, T.A., Gaffrey, M.J., Trejo, J.B., Sagendorf, T.J., Attah, I.K., Moore, R.J., Douglas, C.M., et al. (2024). Signatures of cysteine oxidation on muscle structural and contractile proteins are associated with physical performance and muscle function in older adults: Study of Muscle, Mobility and Aging (SOMMA). Aging Cell *23*, e14094. https://doi.org/10.1111/acel.14094.
13. Day, N.J., Gaffrey, M.J., and Qian, W.-J. (2021). Stoichiometric Thiol Redox Proteomics for Quantifying Cellular Responses to Perturbations. Antioxidants *10*, 499. https://doi.org/10.3390/antiox10030499.
14. Burger, N., and Chouchani, E.T. (2024). A new era of cysteine proteomics – Technological advances in thiol biology. Curr. Opin. Chem. Biol. *79*, 102435. https://doi.org/10.1016/j.cbpa.2024.102435.
15. Cobley, J.N., Margaritelis, N.V., Chatzinikolaou, P.N., Nikolaidis, M.G., and Davison, G.W. (2024). Ten "Cheat Codes" for Measuring Oxidative Stress in Humans. Antioxidants *13*, 877. https://doi.org/10.3390/antiox13070877.
16. Joliffe, I. (2002). Principal Component Analysis. Phil. Trans. Math. Phys. Eng. Sci. *374*, 20150202.
17. Kleinberg, J. (2002). An Impossibility Theorem for Clustering. Adv. Neural Inf. Process. Syst. *15*.
18. Xiao, H., Jedrychowski, M.P., Schweppe, D.K., Huttlin, E.L., Yu, Q., Heppner, D.E., Li, J., Long, J., Mills, E.L., Szpyt, J., et al. (2020). A Quantitative Tissue-Specific Landscape of Protein Redox Regulation during Aging. Cell *180*, 968–983.e24. https://doi.org/10.1016/j.cell.2020.02.012.
19. Cobley, J.N. (2025). Information, Geometry, and Chaos: Revealing Latent Cysteine Butterflies on Fractal Redox Shapes in the Proteomic Spectra. Preprint at Preprints. https://doi.org/10.20944/preprints202507.2120.v1.
20. Cobley, J.N., Chatzinikolaou, P.N., and Schmidt, C.A. (2025). The Nonlinear Cysteine Redox Dynamics in the i-Space: A Proteoform-centric Theory of Redox Regulation. Redox Biol. *81*, 103523. https://doi.org/10.1016/j.redox.2025.103523.
21. Cobley, J.N. (2023). Oxiforms: Unique cysteine residue- and chemotype-specified chemical combinations can produce functionally-distinct proteoforms. Bioessays *45*, 2200248. https://doi.org/10.1002/bies.202200248.
22. Cobley, J.N. (2026). Proteoforms as the true units of physiological function. Eur. J. Appl. Physiol. *126*, 1245–1259. https://doi.org/10.1007/s00421-025-06096-3.
23. Cobley, J.N. (2024). Exploring the unmapped cysteine redox proteoform landscape. Am. J. Physiol. Cell Physiol. *327*, C844–C866. https://doi.org/10.1152/ajpcell.00152.2024.
24. Jones, D.P., and Sies, H. (2015). The Redox Code. Antioxid Redox Sign *23*, 734–746. https://doi.org/10.1089/ars.2015.6247.
25. Held, J.M. (2020). Redox Systems Biology: Harnessing the Sentinels of the Cysteine Redoxome. Antioxid Redox Sign *32*, 659–676. https://doi.org/10.1089/ars.2019.7725.
26. Parvez, S., Long, M.J.C., Poganik, J.R., and Aye, Y. (2018). Redox Signaling by Reactive Electrophiles and Oxidants. Chem. Rev. *118*, 8798–8888. https://doi.org/10.1021/acs.chemrev.7b00698.
27. Prigogine, I. (2009). Dissipative structures, dynamics and entropy. Int. J. Quantum Chem. *9*, 443–456. https://doi.org/10.1002/qua.560090854.
28. Prigogine, I. (1978). Time, Structure, and Fluctuations. Science *201*, 777–785. https://doi.org/10.1126/science.201.4358.777.
29. Ivancevic, V.G., and Ivancevic, T.T. (2011). Ricci flow and nonlinear reaction–diffusion systems in biology, chemistry, and physics. Nonlinear Dyn. *65*, 35–54. https://doi.org/10.1007/s11071-010-9872-6.
30. Cobley, J.N. (2025). Modal Geometry Governs Proteoform Dynamicspreprint at arXiv. https://doi.org/10.48550/arxiv.2508.20004.
31. Stanley, R.P. (2011). Enumerative Combinatorics 2nd Ed. (Cambridge University Press). https://doi.org/10.1017/cbo9781139058520.
32. Smith, L.M., Kelleher, N.L., , et al.; Consortium for Top Down Proteomics, Goodlett, D., Langridge-Smith, P., Goo, Y.A., Safford, G., Bonilla*, L., Kruppa, G., Zubarev, R. (2013). Proteoform: a single term describing protein complexity. Nat. Methods *10*, 186–187. https://doi.org/10.1038/nmeth.2369.
33. Smith, L.M., and Kelleher, N.L. (2018). Proteoforms as the next proteomics currency. Science *359*, 1106–1107. https://doi.org/10.1126/science.aat1884.
34. Jungblut, P.R., Holzhütter, H.G., Apweiler, R., and Schlüter, H. (2008). The speciation of the proteome. Chem. Cent. J. *2*, 16. https://doi.org/10.1186/1752-153x-2-16.
35. Cobley, J.N., Noble, A., and Guille, M. (2026). Cleland immunoblotting quantifies cysteine proteoform ensembles. Free Radic. Biol. Med. *251*, 173–181. https://doi.org/10.1016/j.freeradbiomed.2026.04.026.
36. Brugallé, E., and Shaw, K. (2013). A bit of tropical geometrypreprint at arXiv. https://doi.org/10.48550/arxiv.1311.2360.
37. Litvinov, G., Maslov, V., and Sergeev, S. (2007). Idempotent and tropical mathematics and problems of mathematical physics (Volume I).preprint at arXiv. https://doi.org/10.48550/arxiv.0710.0377.
38. Butkovič, P. (2003). Max-algebra: the linear algebra of combinatorics? Linear Algebra. Appl *367*, 313–335. https://doi.org/10.1016/s0024-3795(02)00655-9.
39. Shannon, C.E. (1948). A Mathematical Theory of Communication. Bell Syst. Tech. J. *27*, 379–423. https://doi.org/10.1002/j.1538-7305.1948.tb01338.x.
40. Halliwell, B. (2006). Oxidative stress and neurodegeneration: where are we now? J. Neurochem. *97*, 1634–1658. https://doi.org/10.1111/j.1471-4159.2006.03907.x.
41. Urrutia, P.J., and Bórquez, D.A. (2023). Expanded bioinformatic analysis of Oximouse dataset reveals key putative processes involved in brain aging and cognitive decline. Free Radic. Biol. Med. *207*, 200–211. https://doi.org/10.1016/j.freeradbiomed.2023.07.018.
42. Cobley, J.N. (2018). Synapse Pruning: Mitochondrial ROS with Their Hands on the Shears. Bioessays *40*, 1800031. https://doi.org/10.1002/bies.201800031.
43. Behring, J.B., van der Post, S., Mooradian, A.D., Egan, M.J., Zimmerman, M.I., Clements, J.L., Bowman, G.R., and Held, J.M. (2020). Spatial and

CellPress
OPEN ACCESS

temporal alterations in protein structure by EGF regulate cryptic cysteine oxidation. Sci. Signal. *13*, eaay7315. https://doi.org/10.1126/scisignal.aay7315.

44. Cobley, J.N., Sakellariou, G.K., Husi, H., and McDonagh, B. (2019). Proteomic strategies to unravel age-related redox signalling defects in skeletal muscle. Free Radic. Biol. Med. *132*, 24–32. https://doi.org/10.1016/j.freeradbiomed.2018.09.012.

45. Li, X., Day, N.J., Feng, S., Gaffrey, M.J., Lin, T.-D., Paurus, V.L., Monroe, M.E., Moore, R.J., Yang, B., Xian, M., and Qian, W.J. (2021). Mass spectrometry-based direct detection of multiple types of protein thiol modifications in pancreatic beta cells under endoplasmic reticulum stress. Redox Biol. *46*, 102111. https://doi.org/10.1016/j.redox.2021.102111.

46. Brown, G.C. (2024). Bioenergetic myths of energy transduction in eukaryotic cells. Front. Mol. Biosci. *11*, 1402910. https://doi.org/10.3389/fmolb.2024.1402910.

47. Cobley, J.N. (2026). A tropical geometry for bounded biochemical state spacespreprint at arXiv. https://doi.org/10.48550/arxiv.2601.13211.

48. Cobley, J. (2026). An Axiomatic Theory of Proteoform Identity Dynamics on Discrete Modal Latticespreprint at SSRN. https://doi.org/10.2139/ssrn.6113577.

49. Lorenz, E.N. (1963). Deterministic Nonperiodic Flow. J. Atmos. Sci. *20*, 130–141. https://doi.org/10.1175/1520-0469(1963)020<0130:dnf>2.0.co;2.

50. Cobley, J.N. (2026). Oxi-Shapes. Zenodo. https://doi.org/10.5281/zenodo.18411792.

Patterns, Volume *7*

Supplemental information

A tropical geometry for bounded biochemical state spaces

James N. Cobley

Supplemental Notes

Supplemental Note 1 — Formal structure of bounded biochemical state spaces

S1.1. State space and observables

Let a biochemical modification state be represented by a bounded occupancy

$$x \in \mathcal{X} := [0,1],$$

where $x = 0$denotes fully unmodified (e.g., fully reduced) and $x = 1$ denotes fully modified (e.g., fully oxidised). For proteoform-level objects comprising $r$sites, we consider the product space

$$\mathcal{X}^{(r)} := [0,1]^r.$$

In many PTM contexts, the measured quantity is an ensemble occupancy (fraction modified), and therefore a coarse-grained observable. We model this via a measurement map

$$M: \Omega \to \mathcal{X},$$

from a (typically unobserved) space of micro-trajectories Ω to a bounded observable x.

S1.2. Hard bounds and closure

The state space $\mathcal{X}$ is closed and bounded. Any operation interpreted as a "translation" $x \mapsto x + \delta$with $\delta \neq 0$ is not closed on $\mathcal{X}$ in general, since $x + \delta \notin [0,1]$ whenever x is sufficiently close to a boundary.

A natural way to represent finite changes while preserving closure is by *saturated* (clipped) maps. For example, define oxidation and reduction updates of magnitude $a, b \geq 0$ by

$$\mathrm{Ox}_a(x) := \min(1, x + a), \mathrm{Red}_b(x) := \max(0, x - b).$$

Then $\mathrm{Ox}_a, \mathrm{Red}_b: \mathcal{X} \to \mathcal{X}$ are well-defined endomorphisms.

S1.3. Absorbing boundaries

Definition (absorbing boundary). A boundary point $x^\star \in \{0,1\}$ is absorbing for an update family $\{F_\theta\}$ if for all admissible θ in a direction,

$$F_\theta(x^\star) = x^\star.$$

Proposition S1 (absorption under saturation). For all $a \geq 0$and $b \geq 0$,

$$\mathrm{Ox}_a(1) = 1, \mathrm{Red}_b(0) = 0.$$

Hence $1$is absorbing for oxidation updates and 0 is absorbing for reduction updates.

Proof. Immediate from the definitions: $\min(1,1+a)=1$and $\max(0,0-b)=0$. □

S1.4. Many-to-one mapping and non-invertibility

Proposition S2 (many-to-one collapse). For any $a>0$,

$$\mathrm{Ox}_a(x)=1 \text{for all } x\in[1-a,1].$$

Similarly, for any $b>0$,

$$\mathrm{Red}_b(x)=0 \text{for all } x\in[0,b].$$

Proof. If $x\geq 1-a$, then $x+a\geq 1$ so $\min(1,x+a)=1$. The reduction case is analogous. □

Corollary S2.1 (non-invertibility). For any $a>0$, Ox_a is not injective (hence not invertible) on $\mathcal{X}$. Likewise, Red_b for any $b>0$.

This formalises the claim that distinct biochemical histories can collapse onto the same observed boundary state.

S1.5. Equal-magnitude changes encode unequal meaning (position dependence)

For a fixed increment $\delta>0$, the *available capacity* to move upward without saturation at state $x$is $c_+(x):=1-x$; downward is $c_-(x):=x$. A change of magnitude δ is therefore interpreted relative to state-dependent capacity.

Proposition S3 (position dependence). For $\delta>0$, the "same" increment δproduces a saturated outcome iff $x\geq 1-\delta$. Hence, the effect of a δ-update depends on $x$through $c_+(x)$.

This captures formally why a shift $0\to 0.1$and $0.5\to 0.6$ are not equivalent: only the former crosses the absorbing neighbourhood where collapse is common and capacity is minimal.

S1.6. Proteoform ensembles induce intrinsic many-to-one structure

Let r binary sites define a proteoform state space $\{0,1\}^r$. A proteoform ensemble is a distribution p on $\{0,1\}^r$, i.e. $p\in\Delta_{2^r}$(the probability simplex). The measured site occupancies are the marginals

$$m_i=\mathbb{E}_p[s_i], i=1,\dots,r,$$

defining a mapping

$$\pi: \Delta_{2^r} \to [0,1]^r, \pi(p) = (m_1, \ldots, m_r).$$

Proposition S4 (non-invertibility of marginalisation). For $r \geq 2$, the map π is generically many-to-one: distinct proteoform distributions can yield identical marginal occupancies.

Proof (constructive). For $r = 2$, let $p^{(1)}$ place mass $(1/2, 0, 0, 1/2)$ on $(00, 01, 10, 11)$ and let $p^{(2)}$ place mass $(0, 1/2, 1/2, 0)$. Both yield marginals $m_1 = m_2 = 1/2$ but $p^{(1)} \neq p^{(2)}$. The general case follows by embedding this 2-site construction within r sites. □

This shows that many-to-one structure is present even before considering dynamics: it is intrinsic to coarse-grained measurement of proteoform ensembles.

Supplemental Note 2 — Incompatibility of bounded, absorbing state spaces with linear/Euclidean representations

This note formalises the sense in which linear and Euclidean frameworks assume properties (global translation, invertibility, symmetry) that are violated by bounded, absorbing state spaces.

S2.1. Linear structure implies global translation and inverses

A real vector space $(V, +)$ is an abelian group under addition: for every $v \in V$, an additive inverse $-v$ exists with $v + (-v) = 0$, and translation maps $T_u(v) = v + u$ are bijections of V.

By contrast, bounded biochemical state spaces $\mathcal{X} = [0,1]$ do not admit a globally closed translation operation $x \mapsto x + \delta$ for arbitrary $\delta \neq 0$. Any attempt to enforce closure by saturation introduces many-to-one collapse near boundaries (Proposition S2), which destroys bijectivity and therefore invertibility.

S2.2. No globally invertible "translation-like" dynamics on [0,1] with absorption

Proposition S5 (absorption implies non-bijectivity). Let $F: \mathcal{X} \to \mathcal{X}$ be any map such that there exists an interval $I \subseteq \mathcal{X}$ with $F(x) = x^\star$ for all $x \in I$ (collapse to an absorbing state). Then F is not injective and has no inverse on $\mathcal{X}$.

Proof. If $| I | > 1$, pick $x \neq y \in I$; then $F(x) = F(y) = x^\star$, so F is not injective. □

Because absorption/collapse is an intrinsic feature of the object (Supplemental Note 1), any representation that assumes invertible dynamics is categorically mismatched.

S2.3. Euclidean distance is translation-invariant; bounded saturation is not

In Euclidean spaces, distances satisfy translation invariance:

$$\| (v + u) - (w + u) \| = \| v - w \| \; \forall u, v, w.$$

In bounded state spaces with saturation, the effect of applying the "same" increment depends on position (Proposition S3), so translation-invariance fails at the level of dynamics. Consequently, Euclidean interpretations of "equal distances imply equal meaning" are not valid for state evolution on [0,1] in the presence of absorbing boundaries.

Supplemental Note 3 | Tropical algebra as a natural realisation of the requirements of bounded biochemical state spaces

Supplemental Notes 1 and 2 established that bounded biochemical state spaces possess hard boundaries, absorbing states, many-to-one ensemble mappings, and generically non-invertible dynamics, and that these properties are categorically incompatible with linear and Euclidean representations. This note addresses the natural follow-up question: why tropical algebra, rather than other boundary-aware mathematical approaches?

We do not claim that tropical algebra is the unique resolution to the categorical mismatch problem. Rather, we argue that it is a natural realisation of the minimal algebraic requirements imposed by the object, and that this naturalness can be understood on both algebraic and physical grounds.

S3.1. Minimal requirements revisited

From Supplemental Notes 1 and 2, any algebraic framework suitable for bounded biochemical state spaces must:

(i) respect hard boundaries without relying on unconstrained translation;

(ii) accommodate absorbing states and many-to-one mappings without assuming global invertibility;

(iii) represent irreversible, path-dependent transitions without assuming symmetry;

(iv) remain well-defined on discrete state spaces such as proteoform ensembles.

These requirements are imposed by the object itself and are not negotiable for a categorically correct representation.

S3.2. How alternative boundary-aware approaches fare

Several established approaches handle bounded data and are worth considering as alternatives.

Supplementary Table 1.

Approach	(i) Hard boundaries intrinsic	(ii) Non-invertibility preserved	(iii) Discrete state spaces	(iv) Invertibility restored by construction
**Linear / Euclidean** e.g. PCA, linear regression	No	No	No	Yes
**Logit transformation**	Partial*	No	No	Yes

Approach	(i) Hard boundaries intrinsic	(ii) Non-invertibility preserved	(iii) Discrete state spaces	(iv) Invertibility restored by construction
**Beta regression**	Partial*	No	No	Yes
**Compositional data analysis** Aitchison / log-ratio	Partial*	No	No	Yes
**Bounded tropical semiring** this work	Yes	Yes	Yes	No

*These approaches acknowledge that data are bounded but handle this by mapping observations into an unbounded space (e.g. the real line or the simplex with log-ratio coordinates) prior to applying standard linear methods. Boundaries are managed by transformation rather than respected intrinsically. Invertibility is restored by construction, concealing the non-invertibility that is a structural property of the biological object (Proposition S2, S5).

S3.3. Algebraic grounds for the naturalness of tropical algebra

Tropical algebra replaces standard addition with the max operation and standard multiplication with capped addition (min(1, a + b) in the bounded variant used here). These operations satisfy requirements (i)-(iv) directly:

- Boundaries are intrinsic: the tropical operations are defined to be closed on [0,1] without external clamping.

- Absorbing states arise structurally: once a state reaches a boundary, further operations in the saturating direction leave it unchanged, reflecting biological saturation as an algebraic property rather than a post-hoc correction.

- Non-invertibility is preserved: many-to-one collapse at boundaries is a consequence of the operations themselves (consistent with Propositions S2 and S5), not a failure to be corrected.

- Discrete state spaces are handled directly: tropical operations are well-defined on finite sets and do not require smooth manifolds or infinitesimal neighbourhoods.

S3.4. Physical grounds for the naturalness of tropical algebra

Beyond algebraic convenience, two features of tropical algebra correspond to physical principles in a way that alternative approaches do not.

First, the tropical min operation — central to min-plus tropical algebra and implicit in the path-dependence structure of the bounded semiring — is physically consistent with the principle of least action. In physical systems, nature selects the path of minimum cost among admissible trajectories. The tropical min selects the minimum among competing values,

encoding path optimality as an algebraic primitive. This correspondence means the framework resonates with how physical systems actually evolve rather than imposing a representational convenience.

Second, tropical algebra is idempotent under the max operation: a ⊕ a = a. This means applying the same operation twice produces no additional change — which is physically exact for a saturated or boundary-proximal biochemical site. A fully oxidised cysteine subjected to further oxidation remains fully oxidised; a fully phosphorylated residue cannot be further phosphorylated. In linear algebra, this behaviour requires external enforcement (clamping); in tropical algebra it is a structural consequence of the operations. Idempotency therefore encodes the physical reality of saturation without additional assumptions.

Together, these algebraic and physical correspondences support the choice of tropical algebra as a natural realisation of the requirements of bounded biochemical state spaces, without requiring the claim that it is the only such realisation.

Supplemental Note 4 | A bounded tropical semiring for biochemical occupancies

S3.1. Definition (bounded tropical operations)

Let $\mathbb{T}_{[0,1]} := ([0,1], \oplus, \otimes)$ with operations

$$a \oplus b := \max(a, b),$$
$$a \otimes b := \min(1, a + b).$$

(Here ⊗ is *capped addition*, ensuring closure on [0,1].)

S3.2. Algebraic properties

Proposition S6 (closure). For all $a, b \in [0,1]$, $a \oplus b \in [0,1]$ and $a \otimes b \in [0,1]$.

Proof. $\max(a, b) \in [0,1]$. Also $a + b \in [0,2]$ so $\min(1, a + b) \in [0,1]$. □

Proposition S7 (commutativity and associativity). ⊕ and ⊗ are commutative and associative on [0,1].

Proof. Commutativity is immediate. Associativity of ⊕ follows from associativity of max . For ⊗,

$$(a \otimes b) \otimes c = \min(1, \min(1, a + b) + c) = \min(1, a + b + c) = a \otimes (b \otimes c).$$

□

Proposition S8 (identities and absorption). The element 0is the identity for both operations:

$$a \oplus 0 = a, a \otimes 0 = a,$$

and 1is absorbing for $\otimes$:

$$a \otimes 1 = 1.$$

Proof. Immediate from definitions. □

This mirrors biochemical structure: 1acts as saturation (absorbing under further oxidation-like composition).

Proposition S9 (idempotency and non-existence of additive inverses). $\oplus$is idempotent: $a \oplus a = a$. Consequently, $([0,1],\oplus)$ cannot be a group and admits no global inverses compatible with $\oplus$.

Proof. $a \oplus a = \max(a, a) = a$. If $\oplus$ had inverses, idempotency would force $a = 0$for all a, a contradiction. □

This provides a clean algebraic statement of non-invertibility without appealing to dynamics.

Supplemental Note 5 | Order dependence and non-commutativity of biochemical transition sequences

The main text distinguishes commutativity of tropical operations from order dependence of *state transition sequences*. We formalise that distinction here using oxidation/reduction operators on [0,1].

S4.1. State transition operators

Define oxidation and reduction operators as in S1:

$$\mathrm{Ox}_a(x) = \min(1, x + a), \mathrm{Red}_b(x) = \max(0, x - b).$$

These maps are monotone and closed on $[0,1]$.

S4.2. Non-commutativity of mixed transition sequences

Proposition S10 (order dependence). There exist $a, b > 0$ and $x \in [0,1]$ such that

$$(\mathrm{Ox}_a \circ \mathrm{Red}_b)(x) \neq (\mathrm{Red}_b \circ \mathrm{Ox}_a)(x).$$

Proof (constructive). Choose $x = 1 - \varepsilon$ with $0 < \varepsilon < a$, and choose $b > a - \varepsilon$. Then

$$(\mathrm{Ox}_a \circ \mathrm{Red}_b)(x) = \mathrm{Ox}_a(1 - \varepsilon - b) = \min(1, 1 - \varepsilon - b + a),$$

while

$$(\mathrm{Red}_b \circ \mathrm{Ox}_a)(x) = \mathrm{Red}_b(1) = \max(0,1 - b) = 1 - b.$$

With the above choices, these quantities differ (one path saturates before reduction, the other reduces after saturation). □

This shows that although $\oplus$ is commutative, *mixed biochemical update sequences* need not commute because boundaries change the effect of subsequent operations.

S4.3. Path dependence via collapse

Proposition S11 (history erasure near boundaries). For any $a > 0$, the set $[1 - a, 1]$is collapsed by $\mathrm{Ox}_a$to the single value 1. Therefore, trajectories entering this region become indistinguishable under subsequent observations.

This formalises "irretrievable loss of history" at the observation level and explains why ensemble dynamics are physically non-invertible.

Supplemental Note 6 | Mathematical construction of Oxi-Shapes

Bounded biochemical state space

Let $\mathcal{L} = \{1, \ldots, N\}$ denote a finite index set corresponding to invariant molecular identities (here, cysteine residues). For each site $i \in \mathcal{L}$, an experimental measurement assigns a scalar oxidation occupancy

$$x_i \in [0,1],$$

representing the fraction of molecules observed in an oxidised state at that site. The unit interval constitutes a **bounded biochemical state space** with absorbing boundaries at 0 (fully reduced) and 1 (fully oxidised).

Measurement-native object construction

An Oxi-Shape is defined as a mapping

$$f: \mathcal{L} \to [0,1], i \mapsto x_i,$$

assigning a bounded scalar state to each invariant site identity.

To visualise this object, sites are embedded in a discrete lattice index space by assigning each $i \in \mathcal{L}$a unique and invariant pair of coordinates (u_i, v_i), forming a two-dimensional discrete lattice. This embedding is **index-based only** and does not encode biological proximity, similarity, or interaction.

The measured oxidation value x_i is represented as a bounded scalar deformation along a third axis. The resulting object is a **high-dimensional discrete lattice with a bounded scalar field**, fully determined by experimental measurements.

At this stage, the object carries **no topology, adjacency, metric, or relational structure** beyond site identity and bounded state assignment.

Algebraic constraints of bounded state spaces

Because $x_i \in [0,1]$, the state space is not closed under unconstrained linear operations. In particular:

- Translation invariance does not hold.
- Linear combinations can generate invalid states outside [0,1].
- Equal numerical differences do not correspond to equal physical changes near boundaries.
- Global invertibility and symmetry assumptions underlying Euclidean vector spaces are violated.

As a consequence, applying linear projections, Euclidean distances, or variance-maximising operators to bounded biochemical occupancies is **categorically incompatible** with the data-generating process.

Oxi-Shapes therefore adopts a **bounded (tropical) algebraic perspective**, in which admissible operations must respect the absorbing boundaries and non-linearity intrinsic to the state space.

4 Information conservation and state occupancy

In Oxi-Shapes, each site i carries a state variable $x_i$that is **mutable across conditions** yet **conserved at the level of information**: site identity is invariant, and whenever a site is observed it is assigned a well-defined bounded value.

There are no null internal states within the measurement space; absence of information arises only from missing observations, not from the algebra of the state space itself.

Corollary.
This construction enables direct information-theoretic interpretation of biochemical state without logarithmic transforms, binning procedures, or assumptions of unboundedness. Measures of order, disorder, and entropy may therefore be defined directly on the bounded object once a representation consistent with the algebra is established.

S1.5 Scope and non-assumptions

The construction above is intentionally minimal. At this stage, Oxi-Shapes does **not** assume:

- temporal dynamics or trajectories,
- kinetic or mechanistic models,
- neighbourhood relations between sites,

- smoothness or continuity,
- biological interaction networks.

All subsequent geometric, energetic, or relational structures are introduced only after this measurement-native object has been defined.

Supplementary Note 7 | Volume and configurational degeneracy in bounded cysteine state space

Definition of the bounded cysteine state space

Consider a protein system with *N* cysteine sites, each of which can occupy a reduced or oxidised state. In Oxi-Shapes, each site *i* is associated with a bounded scalar oxidation value

$$x_i \in [0,1],$$

representing the fraction of molecules in which that cysteine is oxidised at the time of measurement.

The admissible biochemical state space is therefore bounded and discrete, with each site contributing exactly one scalar degree of freedom. The full configuration of the system at a given observational snapshot is represented by the vector

$$\mathbf{x} = (x_1, x_2, \dots, x_N).$$

Volume as a geometric invariant

The total volume *V* of an Oxi-Shapes object is defined as the arithmetic mean of site-wise oxidation values:

$$V = \frac{1}{N}\sum_{i=1}^{N} x_i \, .$$

Geometrically, this quantity corresponds to the average uplift of the discrete lattice along the bounded oxidation axis. Because each site contributes exactly one bounded scalar, the volume is invariant under lattice permutation and independent of site ordering.

Importantly, no transformation, normalisation, or rescaling is applied beyond the intrinsic unit interval already encoded in the data. By construction, this volume is numerically identical to the arithmetic mean oxidation, but it is here interpreted as a geometric invariant of the lattice.

Internal redox entropy as an order–disorder coordinate

We define **internal redox entropy** as this volume coordinate *V*. This quantity provides a measure of order–disorder on the bounded cysteine lattice:

- **Maximum internal order** occurs when all sites occupy the same boundary state (all 0 or all 1), corresponding to a perfectly flat lattice with minimal configurational freedom.
- **Maximum internal disorder** occurs at intermediate occupancy, where reduced and oxidised states are most evenly distributed.

Crucially, this definition makes no claim about thermodynamic entropy, heat exchange, dissipation, or microscopic irreversibility. Internal redox entropy is a *geometric projection* of configurational structure defined on a bounded biochemical state space at a fixed observational snapshot.

Configurational degeneracy of the mean

While the volume *V* uniquely specifies the mean oxidation, it does **not** uniquely specify the underlying configuration. For a system of *N* binary sites, the number of distinct configurations consistent with a given mean oxidation is given by the binomial coefficient:

$$\Omega(N, k) = \binom{N}{k},$$

where

$$k = N \times V.$$

This quantity measures the **configurational degeneracy** associated with a given volume coordinate. As *V* varies from 0 to 1, the degeneracy exhibits a characteristic inverted-U structure, with:

- minimal degeneracy at the boundaries (*V* = 0 or 1),
- maximal degeneracy at *V* = 0.5.

This structure reflects a fundamental property of bounded binary state spaces and is independent of any biological assumptions.

Interpretation of realised configurations

The observed Oxi-Shapes lattice corresponds to one realised configuration among the Ω admissible configurations consistent with the measured mean oxidation. The lattice visualisation therefore specifies **which configuration occurred**, while the degeneracy curve quantifies **how many alternative configurations were mathematically possible but not realised**.

In this sense, internal redox entropy decomposes naturally into:

- a **real component** (the volume / mean oxidation), and
- a **configurational context** (the degeneracy associated with that mean).

This separation clarifies how small changes in mean oxidation can correspond to disproportionately large changes in the size of the admissible configuration space, particularly near the boundaries of the bounded state space.

Scope and limitations

This construction is explicitly mesoscopic and non-invertible. Multiple biochemical histories may map to the same observed configuration, and no claims are made regarding the dynamics, kinetics, or external entropy production that gave rise to the measured state.

Rather, Supplemental Note 2 establishes a mathematically complete and bounded framework for interpreting cysteine redox measurements as geometric objects with well-defined volume and configurational structure.

Supplemental Note 8 | Laplacian curvature and derived energy functionals on the bounded lattice

Let $\phi \in [0,1]^N$ denote the bounded occupancy field defined on a discrete index lattice of Nsites. A minimal 4-neighbour adjacency is imposed on the lattice, yielding a graph $G = (V, E)$ with $|V| = N$. Let Adenote the adjacency matrix and D the diagonal degree matrix. The (unnormalised) graph Laplacian is defined as

$$L = D - A.$$

We define Laplacian curvature on the lattice as the action of the graph Laplacian on the bounded field,

$$\kappa \equiv L\phi,$$

which constitutes a discrete second-difference operator measuring local variation of the scalar field relative to its neighbours.

A quadratic variation energy functional (Dirichlet energy) is defined by

$$E_D(\phi) \equiv \frac{1}{2} \sum_{(i,j)\in E} (\phi_i - \phi_j)^2 \;=\; \frac{1}{2}\, \phi^\top L\phi.$$

This quantity measures the total squared variation of the field across adjacent lattice indices and may be expressed as a per-node energy density by distributing edge contributions equally to incident nodes.

To encode an explicit ground-state bias at $\phi = 0$, we define a bounded Morse-type potential at each lattice site, parameterised by $a > 0$,

$$V(\phi_i) = (1 - e^{-a\phi_i})^2.$$

The resulting **Morse energy functional** is summarised either as the mean

$$\frac{1}{N}\sum_{i=1}^{N} V(\phi_i)$$

or as its total sum over nodes.

All functionals defined here are derived directly from the measurement-native bounded field $\phi$and depend only on the chosen index adjacency. They are introduced as mathematically well-posed descriptors of variation and energy on a bounded biochemical state space. These quantities do not represent thermodynamic energy, heat exchange, dissipation, or microscopic irreversibility.

Supplemental Note 9 | Site-wise bounded tropical geometry of redox state changes

Let $\phi \in [0,1]^N$denote the vector of site-wise cysteine oxidation occupancies measured for a given condition, where each component corresponds to a single cysteine residue. For two conditions $A$and B (e.g. young and old), define the site-wise change field

$$\Delta = \phi^{(B)} - \phi^{(A)}.$$

For each site i, the initial occupancy $x_{A,i} = \phi_i^{(A)}$defines a starting point in the bounded unit interval, and the pair $(x_{A,i}, \Delta_i)$ uniquely represents the site-wise redox transition between conditions.

Bounded site-wise change space

Because oxidation occupancy is bounded to the unit interval, admissible changes satisfy the hard constraints

$$-x_{A,i} \le \Delta_i \le 1 - x_{A,i}.$$

Hence, all site-wise transitions lie within a closed, triangular region of the (x_A, Δ) plane. This feasible region is determined entirely by the bounded state space and does not depend on assumptions about kinetics, dynamics, or regulation.

This construction is measurement-native: no rescaling, normalisation, or projection is applied. The resulting geometry therefore encodes the complete set of physically admissible site-wise redox transitions directly implied by the data.

Symmetric and asymmetric site decomposition

The global redox state of a sample is defined as the arithmetic mean of site-wise occupancies,

$$\bar{\phi} = \frac{1}{N}\sum_{i=1}^{N} \phi_i \, .$$

By linearity, the difference between global means across conditions is given by

$$\bar{\phi}^{(B)} - \bar{\phi}^{(A)} = \frac{1}{N}\sum_{i=1}^{N} \Delta_i \, .$$

Sites with $\Delta_i = 0$ contribute identically to both conditions and therefore cannot influence the global shift. Consequently, any non-zero global change must arise exclusively from sites exhibiting signed change.

This motivates a geometric decomposition of the lattice into:

- a **symmetric subspace**, defined by sites with $| \Delta_i | \leq \varepsilon$, and
- an **asymmetric subspace**, defined by sites with $| \Delta_i | > \varepsilon$,

where ε is a fixed tolerance reflecting measurement precision. This decomposition is exact and parameter-free aside from the explicit tolerance choice.

Headroom-limited action and signed collapse

Although each site formally has one degree of freedom, any realised change is constrained by its available headroom. For a site with initial oxidation x_A, the maximum admissible oxidation is $1 - x_A$, while the maximum admissible reduction is x_A.

Thus, a realised change represents a signed collapse constrained by these limits. For example, a site at $x_A = 0.4$ may increase by at most 0.6 or decrease by at most 0.4; if a decrease occurs, no further reduction is possible without violating the bounded state space. These constraints impose strict geometric limits on site-wise physical action.

Normalised signed redox change

To enable comparison of site-wise changes across different starting occupancies, a normalised signed redox change is defined

$$a_i = \begin{cases} \dfrac{\Delta_i}{1 - x_{A,i}}, & \Delta_i > 0 \\ \dfrac{\Delta_i}{x_{A,i}}, & \Delta_i < 0 \\ 0, & |\Delta_i| \leq \varepsilon, \end{cases}$$

which yields a bounded coordinate $a_i \in [-1,1]$.

This representation preserves directionality while expressing each realised change as a fraction of the site's available redox freedom. Importantly, a is a representation-only quantity: it does not encode dynamics, rates, or energetic costs, but provides a constraint-respecting geometric descriptor of site-wise redox change.

Summary

Together, the bounded change space (x_A, Δ), the symmetric/asymmetric decomposition, and the normalised signed coordinate $a$provide a complete geometric description of site-wise redox state transitions implied by bounded occupancy measurements. These constructions depend only on the unit-interval constraint and arithmetic structure of the data and therefore constitute an exact, assumption-free geometric encoding of site-wise redox reorganisation.
